# Comparison of TCGA and GENIE genomic datasets for the detection of clinically actionable alterations in breast cancer

**DOI:** 10.1038/s41598-018-37574-8

**Published:** 2019-02-06

**Authors:** Pushpinder Kaur, Tania B. Porras, Alexander Ring, John D. Carpten, Julie E. Lang

**Affiliations:** 1 Department of Surgery, Keck School of Medicine, University of Southern California, Los Angeles, CA 90033 United States; 2 University of Southern California, Norris Comprehensive Cancer Center, Los Angeles, CA 90033 United States; 3 Department of Translational Genomics, University of Southern California, Norris Comprehensive Cancer Center, Los Angeles, CA 90033 United States

## Abstract

Whole exome sequencing (WES), targeted gene panel sequencing and single nucleotide polymorphism (SNP) arrays are increasingly used for the identification of actionable alterations that are critical to cancer care. Here, we compared The Cancer Genome Atlas (TCGA) and the Genomics Evidence Neoplasia Information Exchange (GENIE) breast cancer genomic datasets (array and next generation sequencing (NGS) data) in detecting genomic alterations in clinically relevant genes. We performed an *in silico* analysis to determine the concordance in the frequencies of actionable mutations and copy number alterations/aberrations (CNAs) in the two most common breast cancer histologies, invasive lobular and invasive ductal carcinoma. We found that targeted sequencing identified a larger number of mutational hotspots and clinically significant amplifications that would have been missed by WES and SNP arrays in many actionable genes such as PIK3CA, EGFR, AKT3, FGFR1, ERBB2, ERBB3 and ESR1. The striking differences between the number of mutational hotspots and CNAs generated from these platforms highlight a number of factors that should be considered in the interpretation of array and NGS-based genomic data for precision medicine. Targeted panel sequencing was preferable to WES to define the full spectrum of somatic mutations present in a tumor.

## Introduction

A comprehensive understanding of potentially actionable genomic aberrations in tumor samples is important in guiding precision medicine for clinical decision-making. With the development of next-generation sequencing (NGS) technologies, it is feasible to characterize the individual genomic landscape and to identify disease causal variation for diagnosis and therapy. The recent advances in cancer genomics using targeted enrichment sequencing have reliably identified clinically relevant genomic alterations present in solid tumors^1^. However, the functional significance of these alterations is still unexplored and for most patients with metastatic breast cancer, there is a compelling need for selecting clinically relevant beneficial treatment strategies via the identification of genetic alterations driving tumorigenesis.

Large-scale efforts such as the Catalogue of Somatic Mutations (COSMIC), The Cancer Genome Atlas (TCGA) and American Association for Cancer Research (AACR) Genomics Evidence Neoplasia Information Exchange (GENIE) project were designed to help investigators better understand the impact of somatic mutations in cancer. However, the vast heterogeneity of lesions observed in mutations and copy number alterations (CNAs) varies for different genes and tumor histologies^2–4^. Molecular profiling of somatic mutations is increasingly being used to help select new treatment regimens in metastatic disease, although as yet there is no proven survival advantage for this approach. This is a particular concern since the open-label randomized, controlled SHIVA trial found that the use of molecularly targeted agents outside of their indications does not improve progression-free survival when compared to empirical treatment in heavily pre-treated metastatic patients^5^. Others have noted that genomics has not failed, it is just it its early stages of adoption and that N-of-One designs are necessary to adopt personalized medicine since each tumor has such unique biology^6^. The United States Food and Drug Administration (FDA) has recently approved the NGS-based FoundationOne CDx test that identifies actionable alterations in cancer-related genes and can guide treatment decisions. Likewise, a variety of commercial and academic laboratories engage in NGS, with discussion of results at molecular tumor boards to discuss if findings indicate a druggable treatment target^7–9^. However, several technical issues need to be addressed before implementing NGS results into clinical practice. These include consideration of the downstream molecular analysis of: degraded DNA extracted from formalin-fixed, paraffin-embedded (FFPE) specimens, limited amounts of fresh tissue, the degree of stromal cellularity, and variation in the sequencing depth and capture efficiency. These challenges limit the ability to identify clinically relevant aberrations present in cancer cell subpopulations^7,10,11^. In addition, another challenge arising in the analysis of multiple datasets is to identify consistent and reproducible clinically actionable biomarkers from sequencing technologies across cohorts and laboratory platforms. A comprehensive understanding of the detection of genomic alterations in cancer requires an integrative network framework for the analysis of NGS data.

The objective of our study was to investigate which platform (array versus WES and targeted panel sequencing) was most sensitive in identifying clinically significant genomic alterations using the TCGA and GENIE datasets for non-metastatic breast cancer patients.

## Results

### Comparison of the clinicopathological features of TCGA and GENIE cohorts

The clinical characteristics including age, race, ethnicity, tumor grade and hormone receptor status were compared between TCGA and GENIE breast cancer invasive lobular carcinomas (ILC) and invasive ductal carcinomas (IDC) patients (Table 1). No significant differences were found for the mean age of patients for ILC (p = 0.66) and IDC (p = 0.66) patients in TCGA and GENIE datasets. Tumor grade and hormone receptor information were not available from the GENIE dataset.

**Table 1:** Clinicopathological features of the TCGA and GENIE cohorts.

	TCGA		GENIE		ILC (TCGA versus GENIE) p-value	IDC (TCGA versus GENIE) p-value	ILC (TCGA versus GENIE) q-value	IDC (TCGA versus GENIE) q-value
Histological type	ILC (n = 127), n(%)	IDC (n = 490), n(%))	ILC (n = 248), n(%)	IDC (n = 1724), n(%)				
**Age**								
Mean	62.3	57.4	58.9	53	0.66	0.66	0.693	0.693
18–50	29 (22.8%)	163 (33.3%)	63 (25.4%)	734 (42.6%)	0.6141	****<0.0001	0.6448	0.0001
51–70	60 (47.2%)	242 (49.4%)	142 (57.3%)	859 (49.8%)	0.0798	****<0.0001	0.1815	0.0001
71–90	38 (29.9%)	85 (17.3%)	41 (16.5%)	129 (7.5%)	**0.0033	****<0.0001	0.009	0.0001
Not Available (NA)	0 (0.0%)	0 (0.0%)	2 (0.8%)	2 (0.1%)	0.5509	>0.9999	0.6266	0.4083
**Race**								
White	107 (84.3%)	344 (70.2%)	212 (85.5%)	1320 (76.6%)	0.761	****<0.0001	0.7397	0.0001
Black	9 (7.1%)	63 (12.9%)	8 (3.2%)	134 (7.8%)	0.1147	****<0.0001	0.1957	0.0001
Asian	3 (2.4%)	36 (7.3%)	6 (2.4%)	98 (5.7%)	>0.9999	****<0.0001	0.7583	0.0001
Native American	0 (0.0%)	0 (0.0%)	1 (0.4%)	1 (0.1%)	>0.9999	>0.9999	0.7583	0.4083
Asian Indian or Alaska Native	0 (0.0%)	1 (0.2%)	0 (0.0%)	0 (0.0%)	>0.9999	0.2213	0.7583	0.1251
NA	7 (5.5%)	46 (9.4%)	0 (0.0%)	0 (0.0%)	***0.0005	****<0.0001	0.0023	0.0001
Not Evaluated	1 (0.8%)	0 (0.0%)	0 (0.0%)	0 (0.0%)	0.3387	>0.9999	0.4203	0.4083
Other	0 (0.0%)	0 (0.0%)	7 (2.8%)	48 (2.8%)	0.1006	0.3594	0.1957	0.1887
Unknown	0 (0.0%)	0 (0.0%)	14 (5.6%)	123 (7.1%)	**0.0033	*0.0496	0.009	0.0304
**Ethnicity**								
Hispanic or Latino	6 (4.7%)	17 (3.5%)	15 (6.0%)	99 (5.7%)	0.8129	***0.0001	0.7397	0.0001
Not Hispanic or Latino	106 (83.5%)	393 (80.2%)	191 (77.0%)	1196 (69.4%)	0.1787	****<0.0001	0.271	0.0001
NA	14 (11.0%)	80 (16.3%)	0 (0.0%)	0 (0.0%)	****<0.0001	****<0.0001	0.0007	0.0001
Not Evaluated	1 (0.8%)	0 (0.0%)	0 (0.0%)	0 (0.0%)	0.3387	>0.9999	0.4203	0.4083
Unknown	0 (0.0%)	0 (0.0%)	42 (16.9%)	429 (24.9%)	****<0.0001	****<0.0001	0.0007	0.0001
**Tumor grade**								
*T Stage*								
T1	21 (16.5%)	135 (27.6%)	NA	NA	—	—	—	—
T2	59 (46.5%)	300 (61.2%)	NA	NA	—	—	—	—
T3	46 (36.2%)	30 (6.1%)	NA	NA	—	—	—	—
T4	1 (0.8%)	24 (4.9%)	NA	NA	—	—	—	—
TX	0 (0.0%)	1 (0.2%)	NA	NA	—	—	—	—
*N Stage*								
N0	54 (42.5%)	234 (47.8%)	NA	NA	—	—	—	—
N1	38 (29.9%)	170 (34.7%)	NA	NA	—	—	—	—
N2	13 (10.2%)	53 (10.8%)	NA	NA	—	—	—	—
N3	21 (16.5%)	24 (4.9%)	NA	NA	—	—	—	—
NX	1 (0.8%)	9 (1.8%)	NA	NA	—	—	—	—
*M Stage*								
M0	98 (77.2%)	440 (89.8%)	NA	NA	—	—	—	—
MX	29 (22.8%)	50 (10.2%)	NA	NA	—	—	—	—
**Hormone Status**								
*ER status*								
ER-positive	117 (92.1%)	328 (66.9%)	NA	NA	—	—	—	—
ER-negative	8 (6.3%)	133 (27.1%)	NA	NA	—	—	—	—
Not Evaluated	2 (1.6%)	27 (5.5%)	NA	NA	—	—	—	—
Indeterminate	0 (0.0%)	2 (0.4%)	NA	NA	—	—	—	—
Equivocal	0 (0.0%)	0 (0.0%)	NA	NA	—	—	—	—
*PR status*								
PR-positive	100 (78.7%)	284 (58.0%)	NA	NA	—	—	—	—
PR-negative	24 (18.9%)	176 (35.9%)	NA	NA	—	—	—	—
Not Evaluated	2 (1.6%)	28 (5.7%)	NA	NA	—	—	—	—
Indeterminate	1 (0.8%)	2 (0.4%)	NA	NA	—	—	—	—
Equivocal	0 (0.0%)	0 (0.0%)	NA	NA	—	—	—	—
*HER2 status*								
HER2-positive	9 (7.1%)	82 (16.7%)	NA	NA	—	—	—	—
HER2-negative	71 (55.9%)	243 (49.6%)	NA	NA	—	—	—	—
Not Evaluated	22 (17.9%)	71 (14.5%)	NA	NA	—	—	—	—
Indeterminate	1 (0.8%)	5 (1.0%)	NA	NA	—	—	—	—
Equivocal	24 (18.9%)	83 (16.9%)	NA	NA	—	—	—	—
NA	0 (0.0%)	6 (1.2%)	NA	NA	—	—	—	—

^*^Significant p-value.

### Comparison of the number of mutational hotspots in actionable genes in breast cancer TCGA and GENIE datasets

**Figure 1:**
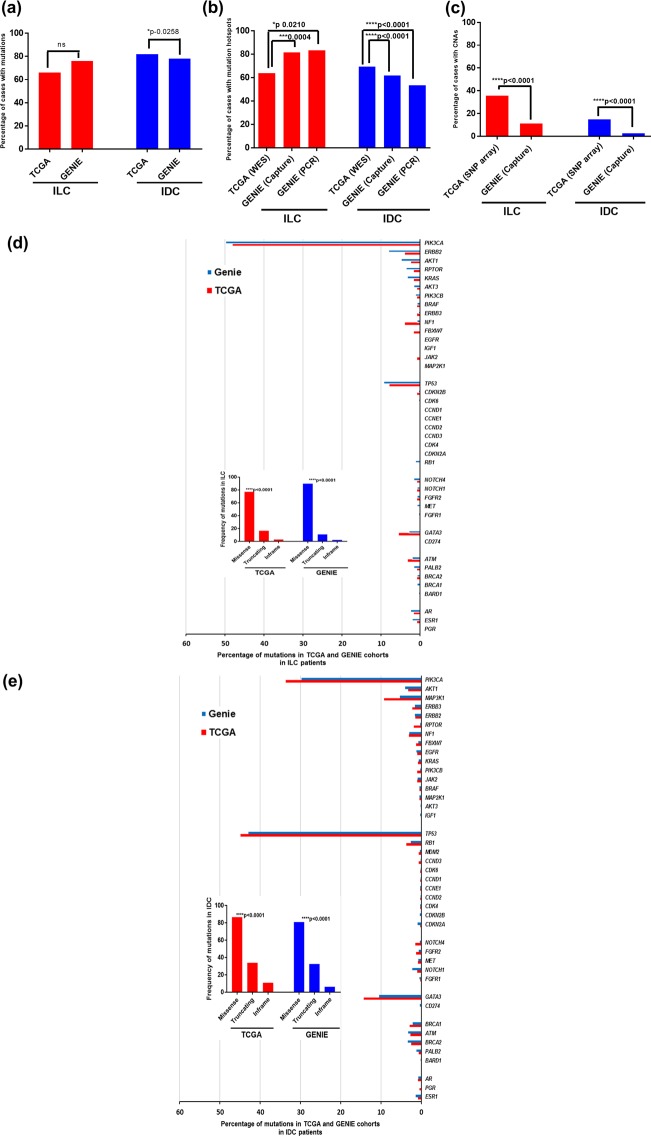
Overview of the genomic alterations in breast cancer patients in the TCGA and GENIE cohort (**a**) Bar graph maps depicting the percentage of cases with mutations obtained from WES (TCGA dataset) versus targeted gene panel (combined data of PCR and hybridization capture, GENIE dataset) approach in 40 actionable genes in ILC and IDC subtypes. (**b**) Bar graph maps depicting the percentage of cases having mutational hotspots obtained from WES (TCGA dataset) versus PCR and hybridization capture (GENIE dataset) in ILC and IDC subtypes (**c**) Bar graph maps depicting the percentage of cases with CNAs obtained from the SNP-based array (TCGA dataset) versus targeted gene panel (hybridization capture, GENIE dataset) approach in 40 actionable genes in ILC and IDC tumors. (**d**) Percentage of mutations in 40 actionable genes in TCGA and GENIE ILC patient samples analyzed by WES versus PCR and hybridization capture technique. PIK3CA dominated the mutational landscape in both data sets and missense mutations (i.e. nontruncating) were more prevalent than truncating and inframe mutations. The inset shows the variation in the percentages of missense, truncating and inframe mutations in the TCGA and GENIE cohort in ILC subtype. (**e**) Percentage of mutations in 40 actionable genes in TCGA and GENIE IDC patients. TP53 was the most commonly mutated gene in TCGA and GENIE IDC patients. The inset shows the variation in the percentages of missense, truncating and inframe mutations in the TCGA and GENIE cohort in IDC tumors. In both cohorts, missense mutations were more prevalent than truncating and inframe mutations in both ILC and IDC tumors (Kruskal-Wallis test, ****p < 0.0001).

Since WES, SNP arrays and targeted gene-panel approaches are routinely used to assess alterations in the coding regions of the genome, we sought to evaluate which of these technologies was more suitable for providing evidence of alterations in actionable targets. Overall, the results showed that there was inconsistency in the genomic alterations (including the percentages of mutational hotspots and CNAs) in the GENIE and TCGA datasets. We also compared the percentage of mutational hotspots between the TCGA and GENIE dataset after stratifying GENIE samples by PCR- and hybridization capture-based approach. The results showed inconsistency in mutational profiles with significant differences in the percentage of identified mutations and CNAs analyzed by WES, PCR and hybridization capture in ILC and IDC cohorts observed. (Fig. 1(a–c)). However, we identified consistency in the mutation frequencies across 40 clinically relevant genes including frequent mutations in PIK3CA, TP53, MAP2K1, NF1 and GATA3 in both of the datasets (Fig. 1(d,e)), which is consistent with previous reports of an association between these gene mutations with breast cancer^12^. Figure 1(d,e) showed the data of all mutations (hotspots and non-hotspots). Hotspot mutations have been annotated with COSMIC database and non-hotspots have been annotated with the Oncology Knowledge Base (OncoKB) and the Clinical Interpretation of Variants in Cancer (CIViC) databases. We applied the Fisher’s exact test to compare the frequencies for all identified mutations. We observed significant differences between the two datasets in some actionable genes such as PIK3CA, ERBB2, TP53, RB1, BRCA2, ESR1, PGR, and ATM, with respect to the number of mutations. To further compare the identified somatic mutations from targeted gene panels to WES, we first assessed the distribution and prevalence of mutations in ILC and IDC samples. The mutations in each gene identified as significant in TCGA dataset were even more prevalent in mutational cluster regions in the GENIE dataset in the IDC subtype. The genes that had a higher number of mutations in the GENIE cohort as compared to TCGA cohort were BRCA2 (57 versus 12, p-0.035 for missense mutations), NOTCH1 (38 versus 5, p-0.04 for missense mutations), and BRCA1 (36 versus 14, p = 0.02 for missense mutations). We also observed 20 mutations in the ESR1 gene in the IDC subtype in the GENIE dataset that were not identified in the same tumor subtype in TCGA. Among these, the 2 main mutations (D538G and E380Q) confer acquired resistance to aromatase inhibitors^13^. In both cohorts, missense mutations were more prevalent than truncating and inframe mutations in both ILC and IDC subtypes (Kruskal-Wallis test, p < 0.0001) (Fig. 1(d,e)). The frequencies, percentages and p-values for missense, truncating and inframe mutations in individual genes in ILC and IDC samples are shown in Supplementary Tables S1 and S2, respectively.

To measure the prevalence of only hotspot mutations in the TCGA and GENIE datasets, we calculated the number of samples in ILC and IDC subtypes that contain =1 and >2 hotspots analyzed by WES and targeted sequencing approach (Supplementary Tables S3 and S4, Supplementary Fig. S1). We found the larger number of mutational hotspots in GENIE than TCGA which may be related to the deeper coverage of the targeted sequencing approach. However, we could not find any significant differences for the percentage of individual mutation hotspot between two datasets. The TCGA cohort had matched normal controls, however, GENIE samples have no matched normal controls. We also searched public databases (COSMIC v87^14^, hotspots.org^15,16^ and 3Dhotspots.org^17^) as references for evaluating whether the identified mutations through WES and targeted sequencing includes any common polymorphisms. We observed that all these hotspots identified in TCGA and GENIE are occurring recurrently in COSMIC database and many of those are present in cancer hotspots database, a resource for statistically significant mutations in cancer^15^. We found many novel hotspots in targeted sequencing data that have been missed through the WES approach (Supplementary Tables S3 and S4, Supplementary Fig. S1) which shows that higher read depth has the potential for higher detection sensitivity of low-level mutations^18,19^. These results demonstrated that target enrichment with higher coverage depths^1^ ranging from ~200x to 4000x permits an in-depth characterization of the genomic landscape to identify rare and low-frequency variants that would have been missed by WES.

### Comparison of copy number calls in actionable genes in breast cancer TCGA and GENIE datasets

**Figure 2:**
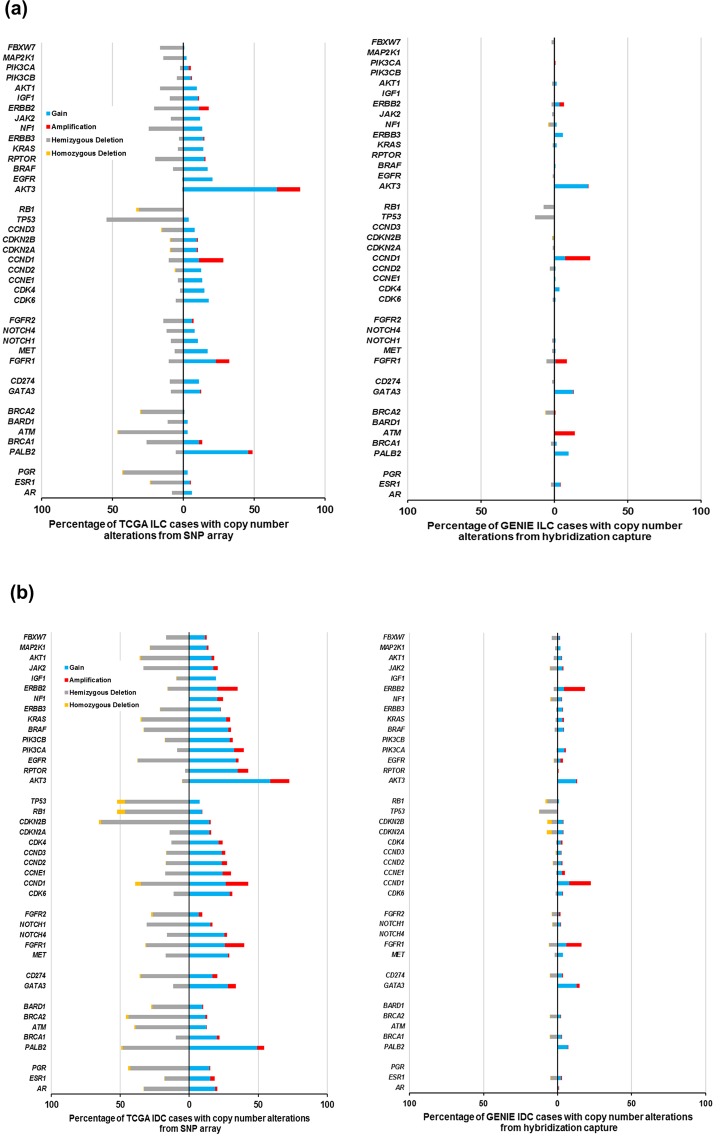
Differential pattern of CNAs in actionable genes in the TCGA and GENIE cohort across ILC and IDC subtypes (**a**) Bars depict the proportion of tumors with CNAs in potentially actionable genes altered in ILC samples. The percentage of tumors with hemizygous deletion (grey), homozygous deletion (yellow), low-level gain (blue) and high-level amplification (red) are shown. (**b**) Bars depict the proportion of tumors with CNAs in potentially actionable genes altered in IDC samples. The percentage of tumors with hemizygous deletion (grey), homozygous deletion (yellow), low-level gain (blue) and high-level amplification (red) are shown.

**Figure 3:**
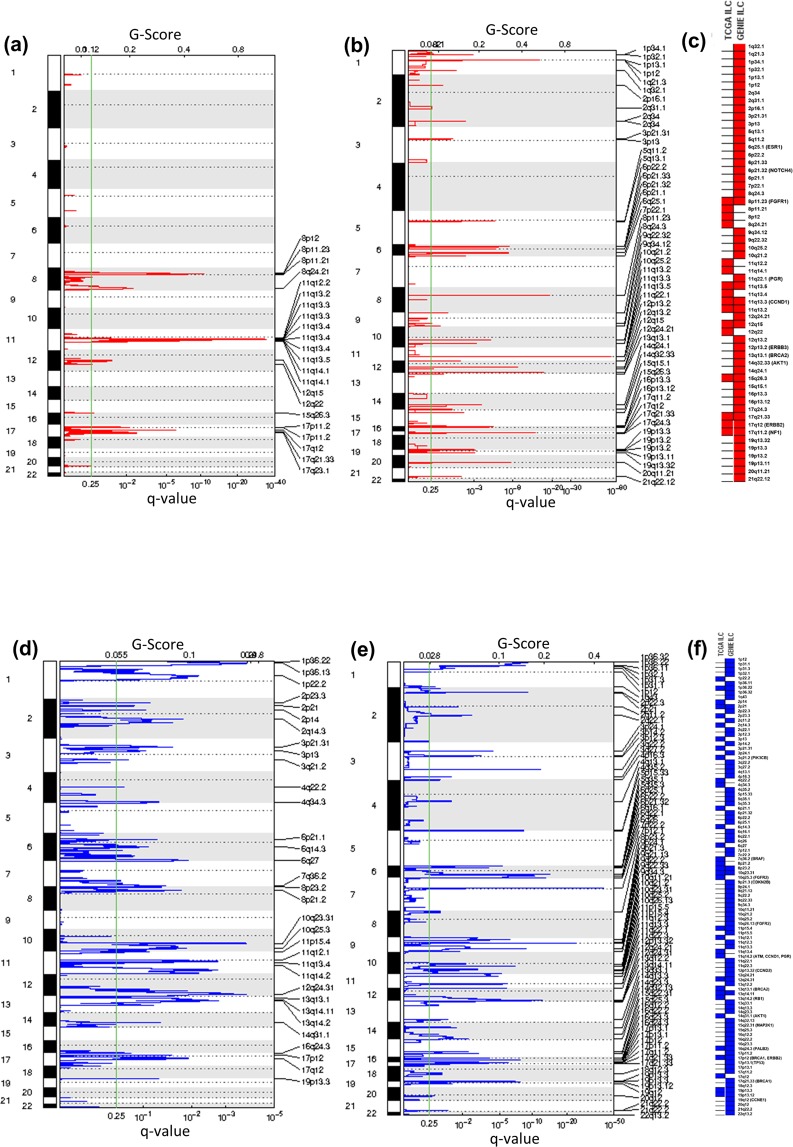
Significant CNAs in ILC cohort in the TCGA and GENIE datasets. (**a**) GISTIC analysis of significant amplifications (red) determined by segmentation analysis from SNP-based array in TCGA ILC cohort. The statistical significance of the aberrations is displayed as FDR (q-values) and scores for each alteration are given at x-axis. The cut-off for significant threshold is 0.25 (green line). The y-axis indicates the chromosome positions and dotted lines indicate the centromeres. (**b**) GISTIC analysis of significant amplifications (red) determined by segmentation analysis from hybridization capture technique in GENIE ILC cohort. (**c**) The heat map represents significant amplified regions in ILC patients in the TCGA and GENIE datasets. The genes from our potential actionable gene list are given in parentheses. (**d**) GISTIC analysis of significant deletions (blue) determined by segmentation analysis from SNP-based array in TCGA ILC cohort. (**e**) GISTIC analysis of significant deletions (blue) determined by segmentation analysis from hybridization capture technique in GENIE ILC cohort. (**f**) The heat map represents significant deleted regions in ILC patients in the TCGA and GENIE datasets. The genes from our potential actionable gene list are given in parentheses.

**Figure 4:**
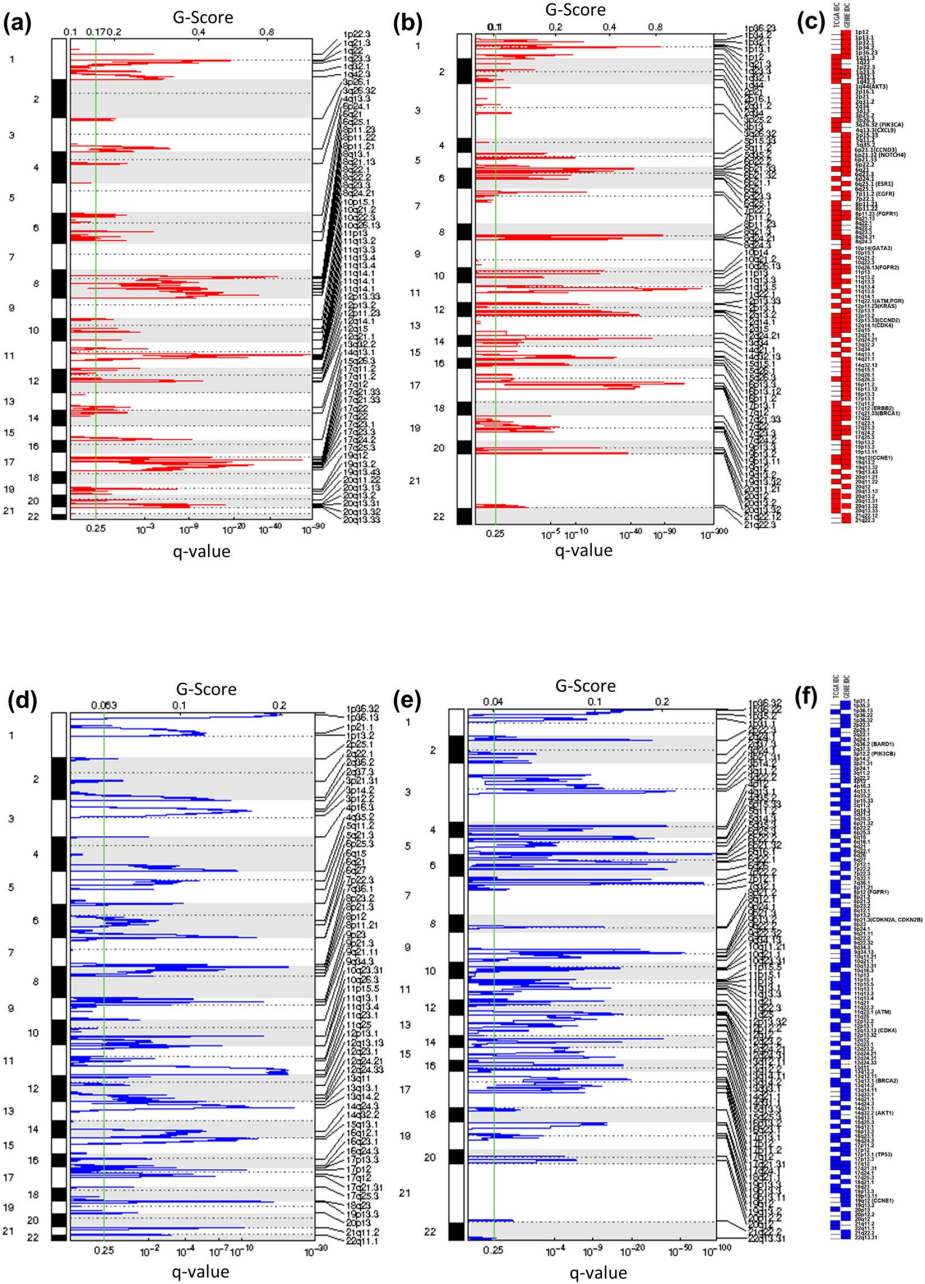
Significant CNAs in IDC cohort in the TCGA and GENIE datasets. (**a**) GISTIC analysis of significant amplifications (red) determined by segmentation analysis from SNP-based array in TCGA IDC cohort. The statistical significance of the aberrations is displayed as false-discovery rate (q-values) and scores for each alteration are given at x-axis. The cut-off for significant threshold is 0.25 (green line). The y-axis indicates the chromosome positions and dotted lines indicate the centromeres. (**b**) GISTIC analysis of significant amplifications (red) determined by segmentation analysis from hybridization capture technique in GENIE IDC cohort. (**c**) The heat map represents significant amplified regions in IDC patients in the TCGA and GENIE datasets. The genes from our potential actionable gene list are given in parentheses. (**d**) GISTIC analysis of significant deletions (blue) determined by segmentation analysis from SNP-based array in TCGA IDC cohort. (**e**) GISTIC analysis of significant deletions (blue) determined by segmentation analysis from hybridization capture technique in GENIE IDC cohort. (**f**) The heat map represents significant deleted regions in IDC patients in the TCGA and GENIE datasets. The genes from our potential actionable gene list are given in parentheses.

The TCGA Pan-Cancer analysis and other studies have shown that CNAs are one of the hallmarks of genomic instability in many cancers and are also the dominant feature in breast cancer^20–23^. A large-scale genomic dataset called the Molecular Taxonomy of Breast Cancer International Consortium (METABRIC) has performed an integrated analysis by combining gene copy number and expression to identify novel biological subgroups^22^. However, a comprehensive understanding of these alterations as putative predictive biomarkers in clinical practice should ultimately facilitate the interpretation of patient data for potential targeted therapy. Thus, the accurate and unbiased identification of recurring CNAs, which are potentially driver events, by using multiple data sets is important to identify the genomic regions of consistent aberration across multiple individuals. We next examined to what extent these two platforms were consistent in detecting actionable genomic CNAs at the sample and gene level. The Fisher’s exact test was used to evaluate the variability in the frequencies of CNA calls. We observed striking differences in the CNA landscape between these two datasets (Fig. 2). The frequencies, percentages and p-values for actionable CNAs in ILC and IDC samples are shown in Supplementary Tables S5 and S6, respectively. The frequency of copy number gain alterations in FGFR1 across ILC samples was 22-fold higher in the TCGA cohort as compared to the GENIE cohort (22% versus 1%, p < 0.0001). RPTOR also harbored frequent copy number gain alterations in 15% of TCGA cases compared to GENIE (0%). We also observed higher frequencies of patients having hemizygous deletions in the hormone receptors in ILC TCGA data set that were not observed in GENIE, including AR (8% versus 0%, p < 0.0001), ESR1 (23% versus 2%, p < 0.0001) and PGR (42% versus 0%, p < 0.0001) (Fig. 2(a)). The differences in the frequencies of copy number amplifications and deletions within actionable genes were also observed in the IDC subtype (Fig. 2(b)). The most frequent actionable alterations in the TCGA IDC dataset in comparison to GENIE were amplification in the regions of 15 genes (AKT3, ESR1, BARD1, BRCA1, PALB2, CD274, GATA3, NOTCH1, NOTCH4, MET, CDK4, CCND3, CCND2, CCNE1, CDK6, p < 0.0001) and deletion in 9 genes (PGR, ATM, BRCA2, BARD1, FGFR1, RB1, BRAF, KRAS, FBXW7, p < 0.05) (Fig. 2(b)). These results indicate that SNP array platforms can detect DNA copy number changes to a reasonable degree of accuracy. We next applied the two-stage linear step-up procedure of Benjamini, Kreiger, and Yekutieli^24^ by setting false-discovery rate (FDR)(Q) to 5% to determine the number of genes with statistically significant different proportion of samples with CNAs between the two datasets. Our comparative analysis for ILC revealed that 34/40 (85%) genes had significant variance in copy number gain, 3/40 (7%) genes in amplification and 34/40 (85%) in hemizygous deletion. Likewise, for IDC, we observed differences in 40/40 (100%) genes for gain, 28/40 (70%) for amplification, 40/40 (100%) genes for hemizygous deletion and 9/40 (22%) genes for homozygous deletion. Since chromosomal aberrations are known to be associated with cancer progression^25,26^, we analyzed amplification and deletions separately to assess which fraction of calls would have been missed by SNP-based array and targeted sequencing approach. We compared both of the datasets for the identification of significant regions of chromosomal amplification and deletions using GISTIC algorithm on the segmented data. The most significant regions (q < 0.25) of copy number amplification in actionable genes were found in GENIE dataset as compared to TCGA dataset in ILC (Fig. 3(a–c)) and IDC cohorts (Fig. 4(a–c)). For deletions, we found common and distinct regions that were deleted in breast cancer-associated genes in both datasets in the ILC (Fig. 3(d–f)) and IDC cohorts (Fig. 4(d–f)). The results of this analysis showed that several potentially important copy number amplifications were capable of being better detected by hybridization capture than SNP-based arrays.

### Comparison of the number of mutational hotspots and copy number calls in actionable genes in NSCLC and colorectal cancer TCGA and GENIE datasets

**Figure 5:**
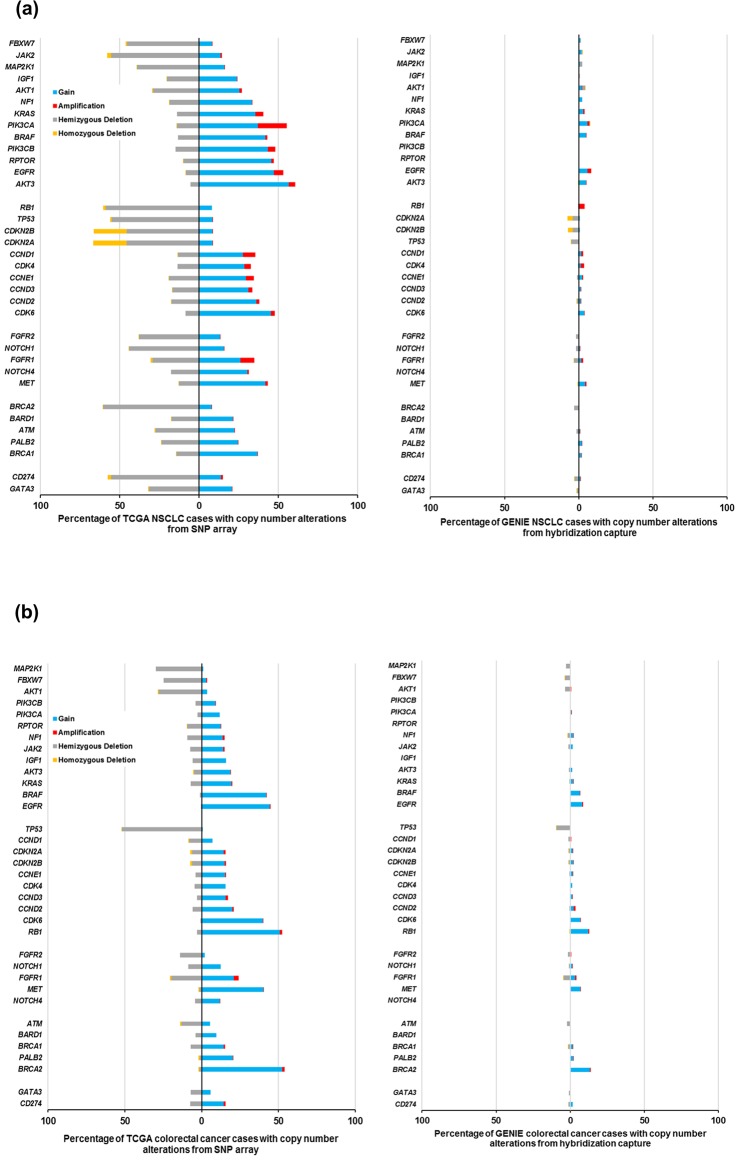
Differential pattern of CNAs in actionable genes in the TCGA and GENIE cohort across NSCLC and colorectal cancer. (**a**) Bars depict the proportion of tumors with CNAs in potentially actionable genes altered in NSCLC samples. (**b**) Bars depict the proportion of tumors with CNAs in potentially actionable genes altered in colorectal cancer samples. The percentage of tumors with hemizygous deletion (grey), homozygous deletion (yellow), low-level gain (blue) and high-level amplification (red) are shown. The Fisher’s exact test was used to determine whether the frequencies of CNAs are different in potentially actionable genes between TCGA and GENIE datasets analyzed by the array and NGS-based technologies.

We further evaluated whether these differences in CNAs were specific for breast cancer or due to tissue preservation methods or platform-specific artifacts. To address this question, we compared the TCGA WES and SNP array data generated from fresh frozen tissues in colorectal^27^ and non-small cell lung cancer (NSCLC)^28^ with the corresponding cancer type in the GENIE targeted panel data obtained from FFPE tissues. We found that there was inconsistency in the frequency distribution of CNAs in both of the data sets for those actionable genes from our list which are considered promising druggable targets for NSCLC, and colorectal cancer, such as KRAS, BRAF, EGFR, ATM, and PIK3CA. In NSCLC alone, we observed higher frequencies of CNAs in many actionable genes in TCGA than in GENIE, such as FGFR1 (9% versus 2%, p < 0.0001) and PIK3CA (18% versus 1%, p < 0.0001) for amplification and CDKN2A (13% versus 0%, p < 0.0001), CDKN2B (20% versus 4%, p < 0.0001) for deletions (Fig. 5(a)). In colorectal cancer, the genes that were significantly enriched for copy number gain in TCGA versus GENIE were BRCA2 (60% versus 23%, p < 0.0001), BRAF (48% versus 11%, p < 0.0001) and KRAS (22% versus 3%, p < 0.0001) (Fig. 5(b)).

In NSCLC and CRC, we observed no significant differences between the proportion of mutations in actionable genes identified through WES and targeted sequencing approach. However, the total number of mutations (including missense, truncating and inframe) in the TP53 gene was greater in GENIE than in TCGA (1709 versus 791, p < 0.0001). Larger number of hotspots and non-hotspots were also detected in GENIE in the NSCLC dataset for genes such as EGFR (738 versus 122, p < 0.0001), NF1 (211 versus 131, p < 0.0001) and PIK3CA (237 versus 94, p-0.038) in comparison to TCGA. Likewise, TP53 was highly enriched for mutations in the GENIE colorectal cancer data as compared to TCGA data (1629 versus 122, p-0.0064). We also observed higher number of mutational hotspots in 3 actionable genes in KRAS (1164 versus 219, p < 0.0001, q-0.0005), EGFR (814 versus 103, p < 0.0001, q-0.0005) and TP53 (1195 versus 499, p < 0.0001, q-0.0005) in GENIE than in TCGA in NSCLC cases.

## Discussion

This study represents an integrated comparison of whole exome, SNP-based array and targeted gene panel sequencing in terms of their ability to detect mutations and CNAs in potentially clinical actionable genes from two-large breast cancer cohort studies. We observed that targeted sequencing is more effective in detecting CNAs than SNP-based array. Although targeted capture sequencing focused on hotspot regions and provided increased quality and reliability at a greater depth in comparison to whole genome sequencing (WGS)^29,30^, it identified only smaller insertions and deletions while ignoring large duplications and deletions^31^. RNA sequencing data was not available from the GENIE dataset and thus it was difficult to determine whether the identified mutational hotspots and gene dosage are related to gene expression. The differences are attributable to the methodology used in both datasets and due to the limited capture design in targeted gene panel and an unequal distribution of targeted sites across the genome that would result in a large number of false positive and false negative calls. These results may be used as a better benchmark for future studies aimed at the identification of actionable alterations from the comparison of large-scale genomic data sets.

We observed that the percent of tumors with CNAs was quite small in GENIE as compared to TCGA, making it difficult to determine the precise spectrum of actionable alterations. The low frequencies in CNAs in these FFPE samples may also be explained due to low input of DNA and degraded DNA that makes the detection procedure complicated for the identification of the regions of deletion. Schweiger *et al*.^32^ have shown that higher sequencing coverage is required for CNA analysis. Although GENIE has also used higher sequencing coverage to detect CNAs, however, there are low frequencies in CNAs in breast cancer, NSCLC and CRC FFPE samples in comparison to TCGA fresh-frozen tissues. Studies have also shown that copy number analysis between the fresh-frozen and FFPE samples varied to a certain degree suggesting that discrepancy in the CNAs frequencies can be due to tissue-preservation methods^33,34^. Another important factor affecting CNA detection is the amount of input DNA that is more than ten-fold higher for the array-based method than sequencing. Thus, the choice of assay and tissue preservation method is important for accurately detecting mutations and CNAs to guide treatment decisions. The MSK-IMPACT tumor profiling assay may distinguish mismatch repair deficiency (MMR-D) and proficient (MMR-P) tumors on the basis of mutational burden in colorectal cancer^35^. The implementation of the results from these platforms in a clinical diagnostic environment requires immunohistochemistry (IHC) validation per multiple guidelines^36–38^. Due to the large variation in detecting genomic alterations between different platforms, many studies have suggested that using multiple computational methods for the identification of genomic alterations reduces the chances of false positive results^39,40^. Recently, Shi *et al*.^41^ identifies that 69% of the mutations from tumor-only WES pipeline were false-positive and even for matched-normal DNA only 36–78% were found consistently in replicate pairs. Since the TCGA cohort is having with or without matched normal controls and GENIE samples have no matched normal controls suggests that caution should be exercised when interpreting these genomic alterations. Torga and colleagues reported very low congruence in tumor-specific genetic alterations for patient-paired samples between the PlasmaSELECT and Guardant360 tests that could lead to different treatment decisions^42^. These results showed that genetic sequencing assays are not always concordant even when the exact same samples are processed, likely due to inherent differences in assay platforms.

From a clinical point of view, our results are of high importance in terms of assessing CNAs from SNP-based array in clinical laboratories, with a particular focus on amplifications in CNAs that would have been missed by this approach. The differences in the CNAs frequency across different platforms would also affect the ability to identify the subtype-specific patterns of alterations (for example, TERT amplification in lung cancer squamous cell carcinoma^43^) and the driver genes that have been mutated by genomic duplication and deletion. Our results highlight some of the issues associated with technical inconsistencies in using molecular profiling for clinical decision-making. NGS technologies continue to evolve with improvements in accuracy along with the rapid production of huge datasets and new methods for identification of recurrent CNAs in multiple samples. However, it is difficult to assess the relative strengths and limitations of different sequencing methods because of the lack of studies that comprehensively compare these technologies. Despite this, variations in the interpretation of copy number changes between the sequencing platforms may become a problem not only for researchers who need to select the method for a dataset of interest, but also a big challenge for clinicians: which platform (array versus NGS) might best detect the underlying genetic driver of the disease in patients? These differences pose a serious challenge when trying to apply these technologies in clinical trials due to the confounding results, which may further impact on treatment decisions for cancer patients. Although both the TCGA and GENIE genomic datasets have CLIA/CAP certifications, validation steps are needed for both the wet and dry bench workflow of NGS-based assays independently by the clinical laboratory before implementation. Furthermore, the platform selection should be based on cross-validating these technologies with more reliable methods such as fluorescence *in situ* hybridization (FISH) and real-time PCR. There is also a need for more specific guidelines to interpret the clinical significance of actionable CNAs detected by array and NGS technologies for improved “genomic-based” therapeutic approaches for cancer patients.

The major limitation of this study is that raw files are not available for the GENIE dataset. In addition, there was much variation in the underlying research strategy of these two datasets such as coverage of the sequencing platforms, different variant calling pipelines and different assays. Differences in the tools/algorithm used in the different steps along with the variant-calling pipelines may also impact the frequency of variants identified. Considering these constraints, we set out to make a comparison demonstrating the frequency of variants using only the processed data as that was available for both datasets through cBioportal.

In conclusion, our study provides an integrated comparison of array and NGS technologies in identifying clinically relevant genomic alterations in potentially actionable genes. We compared the DNA sequencing data between the TCGA and GENIE project to evaluate the concordance in the frequencies of mutations and significant patterns of CNAs in clinically relevant genes in two breast cancer subtypes. Our results showed that SNP array platform identified many candidate regions of CNAs in actionable genes. We found that targeted gene panel sequencing was more effective in detecting a larger number of mutational hotspots and clinically significant duplications and deletions that were missed by WES and SNP-based array. The results of our study may be used as a better benchmark for future studies aimed at the identification of actionable alterations from the comparison of large-scale genomic data sets.

## Methods

### Analysis of potentially breast cancer related genes

For both large-scale genomic datasets, we identified a panel of 49 potentially actionable targets in which biomarkers were linked with FDA-approved or investigational therapeutics in breast cancer studies listed on www.clinicaltrials.gov (Table 2). We analyzed the TCGA^44^ and GENIE^1^ datasets from primary invasive lobular carcinomas (ILC) and invasive ductal carcinomas (IDC) patients for 40 genes from our curated list as 9 genes were not available on the targeted gene panel. Genes were defined as clinically relevant or actionable based on therapeutic and/or diagnostic implications in cancer patients^45^. Our gene panel is not Clinical Laboratory Improvement Amendments (CLIA)/College of American Pathologists (CAP) certified, but the majority of these 49 actionable targets are found in CLIA certified gene panels such as the Memorial Sloan Kettering-Integrated Mutation Profiling of Actionable Cancer Targets (MSK-IMPACT) (410 genes), OncoKB database^46^ (476 cancer-associated genes targeted by FDA-approved drugs or standard therapeutic agents) and Foundation Medicine (315 clinically relevant genes). The intent of our gene panel was to focus on potentially actionable genes with relevance to breast cancer and to maintain a sufficiently focused list in order to permit a detailed comparison of the TCGA and GENIE results as they pertain to clinically relevant gene targets.

**Table 2:** List of potentially breast cancer related genes.

Genes	Foundation One	MSK-IMPACT	OncoKB	Clinical Trials	Candidate Drugs
PIK3CA	P	P	P	NCT02465060, NCT03337724, NCT01513356, NCT01337765, NCT01928459, NCT03243331	Buparlisib, Alpelisib + Fulvestrant, Serabelisib, Copanlisib, GDC-0077, Alpelisib
AKT3	P	P	—	NCT01964924, NCT02162719, NCT01226316, NCT02077569, NCT01277757, NCT02423603, NCT01980277, NCT01964924, NCT01992952	Taselisib + Fulvestrant, Buparlisib + Fulvestrant, Taselisib, GDC-0941
NF1	P	P	P	NCT02465060	Ipatasertib, BKM120, BEZ235, BGJ398 with BYL719, Gedatolisib
PIK3CB	P	P	—	NCT02465060, NCT03337724, NCT01513356, NCT01337765, NCT01928459, NCT03243331	—
RPTOR	—	—	—	NCT02456857, NCT01674140, NCT00107016, NCT02465060, NCT02583542, NCT01390818, NCT01337765	Ipatasertib, AZD5363, PF-04691502, Triciribine, CCT128930
AKT1	P	P	P	NCT01964924, NCT02162719, NCT01226316, NCT02077569, NCT01277757, NCT02423603, NCT01980277, NCT01964924, NCT01992952	Honokiol, AT13148, TIC10 (ONC201), MK2206
FBXW7	P	P	—	—	LY2780301, GSK2141795
IGF1	P	P	—	NCT00984490, NCT02278965, NCT01479179, NCT00984490, NCT00759785, NCT01372618, NCT00897884	—
GRB7	—	—	—	NCT00513292, NCT00004067	LTT462, Binimetinib, BVD523, Trametinib,
KRAS	P	P	P	NCT00894504, NCT02259114, NCT01520389, NCT01337765	MAPK/PI3K/mTOR inhibitors, e.g., MSC1936369B
BRAF	—	P	P	NCT02401347, NCT03065387, NCT01363232, NCT01337765	Everolimus, Temsirolimus
EGFR	P	P	P	NCT02465060, NCT01582191, NCT01934335, NCT01732276, NCT00739063, NCT02720185, NCT00820924, NCT00894504	—
MAP2K1	P	P	P	NCT02322814, NCT01160718, NCT02685657, NCT00147550, NCT01467310, NCT01337765	Buparlisib, Alpelisib + Fulvestrant, Serabelisib, Copanlisib, GDC-0077
JAK2	P	P	P	NCT02041429, NCT02637375, NCT01929941	Alpelisib, Taselisib + Fulvestrant, Buparlisib + Fulvestrant, Taselisib
ERBB2	P	P	P	NCT02465060, NCT03065387, NCT00878709, NCT01953926, NCT00875979	GDC-0941, Ipatasertib, BKM120, BEZ235, BGJ398 with BYL719, Gedatolisib
ERBB3	—	P	—	NCT03065387, NCT00073528, NCT02980341, NCT02297698, NCT01918254, NCT03321981, NCT00073528	—
CCND1	P	P	—	NCT02936206, NCT03304080, NCT01740427, NCT02187783, NCT01037790	Everolimus, AZD8055, Becacizumab, Voxtalisib, PP242
CDKN2A	P	P	P	NCT01740427	OSI-027, Apitolisib, Gedatolisib (PKI-587), Sapanisertib
CDKN2B	P	P	—	NCT01740427	AZD6244, SAR245409, BEZ235
CCND3	P	P	—	NCT02187783	—
CCND2	P	P	—	NCT01037790, NCT00334542, NCT02187783	Ipatasertib, AZD5363, PF-04691502, Triciribine, CCT128930
CCNE1	P	P	—	NCT03184090	Honokiol, AT13148, TIC10 (ONC201), MK2206, LY2780301
CDK6	P	P	—	NCT03184090	GSK2141795
CDK4	P	P	P	NCT03184090	—
TP53	P	P	—	NCT00044993, NCT00004038, NCT01386502, NCT00496860	—
RB1	P	P	—	NCT02599363, NCT03130439, NCT03007979	Tivozanib, AMG 479, Metformin, MK-0646, Pasireotide, Ganitumab
NOTCH4	—	—	—	NCT00645333, NCT01372579	G7–18NATE, NVP-AEW541, BMS-536924, BMS-536924, Dovitinib
NOTCH1	P	P	—	NCT02299635, NCT01208441, NCT00645333, NCT01372579, NCT00106145, NCT01151449, NCT01071564	Cobimetinib, Trametinib, AZD6244, MSC1936369B
ALDH1A1	—	—	—	NCT01190345, NCT01424865, NCT00949013, NCT01688609, NCT02001974, NCT01372579	Selumetinib, PD-325901, GSK1120212, MEK162
MET	P	P	P	NCT02465060, NCT03316586, NCT01837602, NCT01575522, NCT01138384	—
FGFR1	P	P	—	NCT01283945	Cobimetinib, Vemurafenib, Dabrafenib,Trametinib
FGFR2	P	P	—	NCT01283945	BKM120 Plus MEK162, BEZ235 Plus MEK162
WNT1	—	—	—	NCT03243331, NCT01351103	″
ATM	P	P	P	NCT02401347, NCT03344965	Afatinib, Erlotinib, Gefitinib, Osimertinib, Vandetanib, Dasatinib, Lapatinib, Panitumumab
PALB2	P	P	—	NCT02401347, NCT03344965	Cobimetinib, Trametinib, AZD6244, MSC1936369B
BRCA1	P	P	P	NCT02163694, NCT01506609, NCT02032823, NCT03205761, NCT02681562, NCT03150576, NCT02826512, NCT01905592	Selumetinib, PD-325901, GSK1120212, MEK162
BRCA2	P	P	P	NCT02163694, NCT01506609, NCT02032823, NCT03205761, NCT02681562, NCT03150576,	—
BARD1	P	P	—	NCT02826512, NCT01905592	Ruxolitinib, Ganetespib, INCB047986
GATA3	P	P	—	NCT00897065	Ado-trastuzumab emtansine, Lapatinib, Trastuzumab, Pertuzumab, Neratinib
IL4	—	—	—	NCT00039052	Neratinib, GW572016, U3–1402, HER2 vaccine nelipepimut-S
TGFB1	—	—	—	NCT00821964, NCT02538471	Lumretuzumab, MCLA-128, NCT02912949
IL6	—	—	—	NCT03135171, NCT02041429	—
IL15	—	—	—	NCT03175666, NCT03127098	Ribociclib, Palbociclib, Abemaciclib, PD 0332991
CD274	P	P	—	NCT03206203, NCT02447003, NCT02999477, NCT02685059, NCT03430466, NCT02489448, NCT02530489, NCT03430518, NCT03414684, NCT03175666, NCT01042379	Ribociclib, Palbociclib, Abemaciclib
CXCL9	—	—	—	NCT03112590	Ribociclib, Palbociclib, Abemaciclib
ESR1	—	P	P	NCT00849030, NCT03455270, NCT02650817, NCT02734615	Ribociclib, Palbociclib, Abemaciclib
AR	P	P	—	NCT01889238, NCT01918306, NCT02457910, NCT01151046, NCT03207529, NCT02130700, NCT01990209	Ribociclib, Palbociclib, Abemaciclib, PD 0332991
PGR	—	—	—	NCT00849030, NCT01151046, NCT01421472, NCT03241810	Ribociclib, Palbociclib, Abemaciclib
ESR2	—	—	—	NCT00580112, NCT00050427, NCT020898547, NCT02067741	Ribociclib, Palbociclib, Abemaciclib, PD 0332991

P = Present in the gene panel, —= Not present in the gene panel, not present in the clinical trials, not present in the candidate drugs.

### TCGA and GENIE data

We assessed the whole-exome DNA sequencing and Affymetrix SNP 6.0 array data for 127 ILC and 490 IDC from TCGA cohort and compared these with the third data release for GENIE targeted sequencing data for 248 ILC and 1724 IDC cases. The mutations and CNAs generated from Affymetrix array and NGS technologies were retrieved from cBioportal^47,48^. Only GENIE samples that were screened using hybridization-based capture approach, as opposed to PCR-based approach, were analyzed for CNAs. The sample size of this subset of GENIE samples analyzed for CNAs is given in Supplementary Table S7. All patient samples were de-identified and encoded with TCGA and GENIE sample codes. We compared the array and NGS results from TCGA fresh frozen tissues and GENIE FFPE tissues to determine concordance between each platform. For the validation of both datasets, we also compared the TCGA WES and SNP array data generated from fresh frozen tissues in colorectal^27^ and non-small cell lung cancer (NSCLC)^28^ with the corresponding cancer type in the GENIE targeted panel data^1^ obtained from FFPE tissues. We obtained the mutational and CNA events using cBioPortal for array data from TCGA NSCLC (n = 1144) and targeted gene panel sequencing data from GENIE (n = 3694). The mutational and CNA events for colorectal cancer were also obtained from cBioPortal for array data from TCGA colorectal (n = 226) and targeted gene panel sequencing data from GENIE (n = 2574).

### Comparison of DNA mutations from WES and targeted gene panel sequencing data

For the identification of putative hotspots in clinically actionable genes, we downloaded the mutational hotspot data for TCGA and GENIE cohorts using cBioportal from the sequenced exomes of breast cancer patients (based on prespecified classifications or groups). The Fisher’s exact test was used to evaluate the variability in the frequencies of mutations for 40 actionable genes between both data sets for ILC and IDC subtypes. The Kruskal-Wallis test was applied to assess which mutation types are more prevalent in both breast cancer subtypes.

### Comparison of CNAs from SNP-based array and targeted gene panel sequencing data

To determine the copy number status of an individual gene in any given patient, we used copy number datasets within the cBioportal generated by Genomic Identification of Significant Targets in Cancer (GISTIC) algorithms^26^. CNA was characterized by measured copy number (expressed as a log2 ratio), and by the extent of change in the genome. The CNA thresholds were determined according to the set of discrete copy number calls provided by GISTIC: deep loss/homozygous deletion (−2), shallow loss/hemizygous deletion (−1), low-level gain (1), and high-level amplification (2). The copy number data was not available from the patients analyzed by PCR method in GENIE data set. The Fisher’s exact test was used to determine whether the frequencies of CNAs are different in actionable genes between TCGA and GENIE datasets analyzed by the array and NGS-based technologies. The identification of significantly amplified and deleted regions among potentially actionable genes was done using the GISTIC algorithm. The data was aligned to genome build hg19. The algorithm was executed within the Broad Firehose infrastructure. The GISTIC analysis was conducted separately on the ILC and IDC subtypes in TCGA and GENIE breast cancer study.

### Statistical Analysis

Statistical analysis for comparing the mutations and CNAs was performed using GraphPad Prism version 7. The most prevalent mutations among missense, truncating and inframe mutations were calculated using the Kruskal-Wallis test. The Fisher’s exact test was used to calculate the variability for the frequencies of hotspots and CNAs. The two-stage linear step-up procedure of Benjamini, Kreiger and Yekutieli by setting FDR(Q) to 5% was used to correct p-values for multiple testing.

### Ethics approval and consent to participate

This study was performed in strict accordance with the recommendations of data access guidelines of TCGA and AACR project GENIE datasets. We received administrative permission for downloading the restricted-access data for breast cancer patients from the TCGA Data Access Committee (Project # 10345).

## Supplementary information

Supplementary Dataset 1

## Data Availability

The datasets analyzed in the current study are publicly available in cBioportal and sage synapse platform.

## References

[1] AACR Project GENIE: Powering Precision Medicine through an International Consortium. *Cancer discovery* **7**, 818–831, 10.1158/2159-8290.cd-17-0151 (2017). 10.1158/2159-8290.CD-17-0151 PMC5611790 28572459

[2] Mamanova L (2010). Target-enrichment strategies for next-generation sequencing. Nat Methods.

[3] Altmuller J, Budde BS, Nurnberg P (2014). Enrichment of target sequences for next-generation sequencing applications in research and diagnostics. Biol Chem.

[4] Choi M (2009). Genetic diagnosis by whole exome capture and massively parallel DNA sequencing. Proc Natl Acad Sci USA.

[5] Le Tourneau C (2015). Molecularly targeted therapy based on tumour molecular profiling versus conventional therapy for advanced cancer (SHIVA): a multicentre, open-label, proof-of-concept, randomised, controlled phase 2 trial. The Lancet. Oncology.

[6] Wheler JJ (2014). Unique molecular signatures as a hallmark of patients with metastatic breast cancer: implications for current treatment paradigms. Oncotarget.

[7] Frampton GM (2013). Development and validation of a clinical cancer genomic profiling test based on massively parallel DNA sequencing. Nat Biotechnol.

[8] Drilon A (2015). Broad, Hybrid Capture-Based Next-Generation Sequencing Identifies Actionable Genomic Alterations in Lung Adenocarcinomas Otherwise Negative for Such Alterations by Other Genomic Testing Approaches. Clinical cancer research: an official journal of the American Association for Cancer Research.

[9] Villaflor V (2016). Biopsy-free circulating tumor DNA assay identifies actionable mutations in lung cancer. Oncotarget.

[10] Hadd AG (2013). Targeted, high-depth, next-generation sequencing of cancer genes in formalin-fixed, paraffin-embedded and fine-needle aspiration tumor specimens. J Mol Diagn.

[11] Yau C (2010). A statistical approach for detecting genomic aberrations in heterogeneous tumor samples from single nucleotide polymorphism genotyping data. Genome Biol.

[12] Powell E, Piwnica-Worms D, Piwnica-Worms H (2014). Contribution of p53 to metastasis. Cancer Discov.

[13] Jeselsohn R, Buchwalter G, De Angelis C, Brown M, Schiff R (2015). ESR1 mutations-a mechanism for acquired endocrine resistance in breast cancer. Nat Rev Clin Oncol.

[14] Forbes SA (2008). The Catalogue of Somatic Mutations in Cancer (COSMIC). Current protocols in human genetics.

[15] Chang, M. T. *et al*. Accelerating Discovery of Functional Mutant Alleles in Cancer. *Cancer discovery* **8**, 174–183, 10.1158/2159-8290.cd-17-0321https://www.cancerhotspots.org/ (2018). 10.1158/2159-8290.CD-17-0321 PMC5809279 29247016

[16] Chang, M. T. *et al*. Identifying recurrent mutations in cancer reveals widespread lineage diversity and mutational specificity. *Nature biotechnology* **34**, 155–163, 10.1038/nbt.3391https://www.cancerhotspots.org/ (2016) 10.1038/nbt.3391 PMC4744099 26619011

[17] Gao, J. *et al*. 3D clusters of somatic mutations in cancer reveal numerous rare mutations as functional targets. *Genome medicine* **9**, 4, 10.1186/s13073-016-0393-xhttps://www.3dhotspots.org/ (2017). 10.1186/s13073-016-0393-x PMC5260099 28115009

[18] Alioto TS (2015). A comprehensive assessment of somatic mutation detection in cancer using whole-genome sequencing. Nature Communications.

[19] Cai L, Yuan W, Zhang Z, He L, Chou K-C (2016). In-depth comparison of somatic point mutation callers based on different tumor next-generation sequencing depth data. Scientific Reports.

[20] Bignell GR (2010). Signatures of mutation and selection in the cancer genome. Nature.

[21] Ciriello G (2013). Emerging landscape of oncogenic signatures across human cancers. Nat Genet.

[22] Curtis C (2012). The genomic and transcriptomic architecture of 2,000 breast tumours reveals novel subgroups. Nature.

[23] Siegel MB (2018). Integrated RNA and DNA sequencing reveals early drivers of metastatic breast cancer. The Journal of clinical investigation.

[24] Benjamini Y, Yekutieli KA (2006). D. Adaptive linear step-up procedures that control the false discovery rate. Biometrika.

[25] Beroukhim R (2010). The landscape of somatic copy-number alteration across human cancers. Nature.

[26] Mermel CH (2011). GISTIC2.0 facilitates sensitive and confident localization of the targets of focal somatic copy-number alteration in human cancers. Genome biology.

[27] Comprehensive molecular characterization of human colon and rectal cancer. *Nature* **487**, 330–337, 10.1038/nature11252 (2012). 10.1038/nature11252 PMC3401966 22810696

[28] Campbell JD (2016). Distinct patterns of somatic genome alterations in lung adenocarcinomas and squamous cell carcinomas. Nat Genet.

[29] Zhao L (2015). Next-generation sequencing-based molecular diagnosis of 82 retinitis pigmentosa probands from Northern Ireland. Hum Genet.

[30] Tajiguli A (2016). Next-generation sequencing-based molecular diagnosis of 12 inherited retinal disease probands of Uyghur ethnicity. Sci Rep.

[31] Chen Y (2017). SeqCNV: a novel method for identification of copy number variations in targeted next-generation sequencing data. BMC Bioinformatics.

[32] Schweiger MR (2009). Genome-wide massively parallel sequencing of formaldehyde fixed-paraffin embedded (FFPE) tumor tissues for copy-number- and mutation-analysis. PLoS One.

[33] Menon R (2012). Exome enrichment and SOLiD sequencing of formalin fixed paraffin embedded (FFPE) prostate cancer tissue. Int J Mol Sci.

[34] Robbe, P. *et al*. Clinical whole-genome sequencing from routine formalin-fixed, paraffin-embedded specimens: pilot study for the 100,000 Genomes Project. *Genetics in medicine: official journal of the American College of Medical Genetics*, 10.1038/gim.2017.241 (2018). 10.1038/gim.2017.241 PMC6520241 29388947

[35] Stadler ZK (2016). Reliable Detection of Mismatch Repair Deficiency in Colorectal Cancers Using Mutational Load in Next-Generation Sequencing Panels. Journal of clinical oncology: official journal of the American Society of Clinical Oncology.

[36] Teutsch SM (2009). The Evaluation of Genomic Applications in Practice and Prevention (EGAPP) Initiative: methods of the EGAPP Working Group. Genet Med.

[37] Ladabaum U (2011). Strategies to identify the Lynch syndrome among patients with colorectal cancer: a cost-effectiveness analysis. Annals of internal medicine.

[38] Giardiello FM (2014). Guidelines on genetic evaluation and management of Lynch syndrome: a consensus statement by the US Multi-Society Task Force on colorectal cancer. Gastroenterology.

[39] Pinto D (2010). Functional impact of global rare copy number variation in autism spectrum disorders. Nature.

[40] Xu B (2008). Strong association of de novo copy number mutations with sporadic schizophrenia. Nat Genet.

[41] Shi W (2018). Reliability of Whole-Exome Sequencing for Assessing Intratumor Genetic Heterogeneity. Cell Reports.

[42] Torga, G. & Pienta, K. J. Patient-Paired Sample Congruence Between 2 Commercial Liquid Biopsy Tests. *JAMA oncology*, 10.1001/jamaoncol.2017.4027 (2017). 10.1001/jamaoncol.2017.4027 PMC6145681 29242909

[43] Pikor LA, Ramnarine VR, Lam S, Lam WL (2013). Genetic alterations defining NSCLC subtypes and their therapeutic implications. Lung cancer (Amsterdam, Netherlands).

[44] Ciriello G (2015). Comprehensive Molecular Portraits of Invasive Lobular Breast. Cancer. Cell.

[45] Van Allen EM (2014). Whole-exome sequencing and clinical interpretation of formalin-fixed, paraffin-embedded tumor samples to guide precision cancer medicine. Nature medicine.

[46] Chakravarty, D. *et al*. OncoKB: A Precision Oncology Knowledge Base. *JCO Precision Oncology* **1**, 1–16, 10.1200/po.17.00011http://oncokb.org/#/ (2017). 10.1200/PO.17.00011 PMC5586540 28890946

[47] Gao J (2013). Integrative analysis of complex cancer genomics and clinical profiles using the cBioPortal. Sci Signal.

[48] Cerami E (2012). The cBio cancer genomics portal: an open platform for exploring multidimensional cancer genomics data. Cancer Discov.

