# Supplementary material for: Comparison of TCGA and GENIE genomic datasets for the detection of clinically actionable alterations in breast cancer
Source: Sci Rep. 2019 Feb 6;9:1482. doi: 10.1038/s41598-018-37574-8 (PMC6365517; doi:10.1038/s41598-018-37574-8)
Supplement: Supplementary file 1 — Supplementary Dataset 1 [file 41598_2018_37574_MOESM1_ESM.pdf]

Comparison of TCGA and GENIE genomic datasets for the detection of clinically actionable alterations in breast cancer

Pushpinder Kaur[1,2], Tania B. Porras[1,2], Alexander Ring[1,2], John D. Carpten[2,3] and Julie E. Lang[1,2*]

[1]Department of Surgery, Keck School of Medicine, University of Southern California, Los Angeles, CA, 90033, United States

[2]University of Southern California, Norris Comprehensive Cancer Center, Los Angeles, CA, 90033, United States

[3]Department of Translational Genomics, University of Southern California, Norris Comprehensive Cancer Center, Los Angeles, CA, 90033, United States

***Corresponding Author:**

Julie E. Lang, M.D, FACS
Associate Professor, Department of Surgery,
Director, Breast Cancer Research Program
University of Southern California (USC) Norris Comprehensive Cancer Center
1510 San Pablo Street, Suite 514
Los Angeles, CA 90033
(T) - 323.865.3535
(E) – julie.lang@med.usc.edu

Supplementary Table S1: Frequencies, percentages and p-values of missense, truncating and inframe mutations in ILC in the TCGA and GENIE cohorts

Genes	Missense mutations (Frequency, percentage) in TCGA cohort	Missense mutations (Frequency, percentage) in GENIE cohort	Missense mutations p-value (fisher's exact test)	Missense mutations q-value	Truncating mutations (Frequency, percentage) in TCGA cohort	Truncating mutations (Frequency, percentage) in GENIE cohort	Truncating mutations p-value (fisher's exact test)	Truncating mutations q-value	Inframe mutations (Frequency, percentage) in TCGA cohort	Inframe mutations (Frequency, percentage) in GENIE cohort	Inframe mutations p-value (fisher's exact test)	Inframe mutations q-value
**PIK3CA**	**59, 46.5**	**120, 48.4**	**0.7**	**1.00**	**0, 0.0**	**0, 0.02**	**1.0**	**1.03**	**2, 1.57**	**4, 1.61**	**1.04**	**1.05**
ERBB2	5, 3.9	18, 7.3	0.3	1.00	0, 0.0	0, 0.0	1.0	1.0	0, 0.00	2, 0.80	1.0	1.0
AKT1	3, 2.4	12, 4.8	0.4	1.00	0, 0.0	0, 0.0	1.0	1.0	0, 0.00	0, 0.00	1.0	1.0
RPTOR	2, 1.6	8, 3.2	0.5	1.00	0, 0.0	1, 0.4	1.0	1.0	0, 0.00	0, 0.00	1.0	1.0
KRAS	2, 1.6	5, 2.0	1.0	1.00	0, 0.0	3, 1.2	0.6	1.0	0, 0.00	0, 0.00	1.0	1.0
AKT3	1, 0.8	4, 1.6	0.7	1.00	0, 0.0	0, 0.0	1.0	1.0	0, 0.00	0, 0.00	1.0	1.0
PIK3CB	1, 0.8	3, 1.2	1.0	1.00	0, 0.0	0, 0.0	1.0	1.0	0, 0.00	0, 0.00	1.0	1.0
BRAF	1, 0.8	2, 0.8	1.0	1.00	0, 0.0	0, 0.0	1.0	1.0	0, 0.00	0, 0.00	1.0	1.0
ERBB3	1, 0.8	1, 0.4	1.0	1.00	0, 0.0	0, 0.0	1.0	1.0	0, 0.00	0, 0.00	1.0	1.0
NF1	1, 0.8	1, 0.4	1.0	1.00	**4, 3.15**	**1, 0.40**	***0.0468**	1.0	0, 0.00	0, 0.00	1.0	1.0
FBXW7	1, 0.8	0, 0.0	0.3	1.00	1, 0.79	0, 0.0	0.3	1.0	0, 0.00	0, 0.00	1.0	1.0
EGFR	0, 0.0	0, 0.0	1.0	1.00	0, 0.0	0, 0.0	1.0	1.0	0, 0.00	0, 0.00	1.0	1.0
IGF1	0, 0.0	0, 0.0	1.0	1.00	0, 0.0	0, 0.0	1.0	1.0	0, 0.00	0, 0.00	1.0	1.0
JAK2	0, 0.0	0, 0.0	1.0	1.00	1, 0.78	0, 0.0	0.3	1.0	0, 0.00	0, 0.00	1.0	1.0
MAP2K1	0, 0.0	0, 0.0	1.0	1.00	0, 0.00	0, 0.0	1.0	1.0	0, 0.00	0, 0.00	1.0	1.0
TP53	7, 5.5	16, 6.5	0.8	1.00	3, 2.36	7, 2.82	1.0	1.0	0, 0.00	0, 0.00	1.0	1.0
CDKN2B	1, 0.8	0, 0.0	1.0	1.00	0, 0.0	0, 0.0	1.0	1.0	0, 0.00	0, 0.00	1.0	1.0
CDK6	0, 0.0	0, 0.0	1.0	1.00	0, 0.0	0, 0.0	1.0	1.0	0, 0.00	0, 0.00	1.0	1.0
CCND1	0, 0.0	1, 0.4	1.0	1.00	0, 0.0	0, 0.0	1.0	1.0	0, 0.00	0, 0.00	1.0	1.0
CCNE1	0, 0.0	0, 0.0	1.0	1.00	0, 0.0	0, 0.0	1.0	1.0	0, 0.00	0, 0.00	1.0	1.0
CCND2	0, 0.0	0, 0.0	1.0	1.00	0, 0.0	0, 0.0	1.0	1.0	0, 0.00	0, 0.00	1.0	1.0
CCND3	0, 0.0	0, 0.0	1.0	1.00	0, 0.0	0, 0.0	1.0	1.0	0, 0.00	0, 0.00	1.0	1.0
CDK4	0, 0.0	0, 0.0	1.0	1.00	0, 0.0	0, 0.0	1.0	1.0	0, 0.00	0, 0.00	1.0	1.0
CDKN2A	0, 0.0	1, 0.4	1.0	1.00	0, 0.0	0, 0.0	1.0	1.0	0, 0.00	0, 0.00	1.0	1.0
RB1	0, 0.0	0, 0.0	1.0	1.00	0, 0.0	3, 1.20	0.6	1.0	0, 0.00	0, 0.00	1.0	1.0
NOTCH4	1, 0.8	2, 0.8	1.0	1.00	0, 0.0	0, 0.0	1.0	1.0	0, 0.00	0, 0.00	1.0	1.0
NOTCH1	1, 0.8	4, 1.6	0.7	1.00	0, 0.0	0, 0.0	1.0	1.0	0, 0.00	0, 0.00	1.0	1.0
FGFR2	1, 0.8	1, 0.4	1.0	1.00	0, 0.0	0, 0.0	1.0	1.0	0, 0.00	0, 0.00	1.0	1.0
MET	0, 0.0	2, 0.8	1.0	1.00	0, 0.0	0, 0.0	1.0	1.0	0, 0.00	0, 0.00	1.0	1.0
FGFR1	0, 0.0	2, 0.8	0.6	1.00	0, 0.0	0, 0.0	1.0	1.0	0, 0.00	0, 0.00	1.0	1.0
GATA3	2, 1.6	1, 0.4	0.3	1.00	5, 3.93	6, 2.41	0.5	1.0	0, 0.00	0, 0.00	1.0	1.0
CD274	0, 0.0	0, 0.0	1.0	1.00	0, 0.00	0, 0.00	1.0	1.0	0, 0.00	0, 0.00	1.0	1.0
ATM	3, 2.4	3, 1.2	0.4	1.00	1, 0.78	2, 0.80	1.0	1.0	0, 0.00	0, 0.00	1.0	1.0
PALB2	1, 0.8	3, 1.2	1.0	1.00	0, 0.0	1, 0.40	1.0	1.0	0, 0.00	0, 0.00	1.0	1.0
BRCA2	1, 0.8	1, 0.4	1.0	1.00	0, 0.0	1, 0.40	1.0	1.0	0, 0.00	0, 0.00	1.0	1.0
BRCA1	0, 0.0	1, 0.4	1.0	1.00	0, 0.0	1, 0.40	1.0	1.0	0, 0.00	0, 0.00	1.0	1.0
BARD1	0, 0.0	1, 0.4	1.0	1.00	0, 0.0	0, 0.0	1.0	1.0	0, 0.00	0, 0.00	1.0	1.0
AR	1, 0.8	6, 2.4	0.4	1.00	0, 0.0	0, 0.0	1.0	1.0	1, 0.78	0, 0.00	1.0	1.0
ESR1	1, 0.8	4, 1.6	0.7	1.00	0, 0.0	1, 0.40	1.0	1.0	0, 0.00	0, 0.00	1.0	1.0
PGR	0, 0.0	0, 0.0	1.0	1.00	0, 0.0	0, 0.0	1.0	1.0	0, 0.00	0, 0.00	1.0	1.0
	***- significant p-value.**											

Supplementary Table S2: Frequencies, percenatges and p-values of missense, truncating and inframe mutations in IDC in the TCGA and GENIE cohorts

Genes	Missense mutations (Frequency, percentage) in TCGA cohort	Missense mutations (Frequency, percentage) in GENIE cohort	Missense mutations p-value (chi-square test)	Missense mutations q-value	Truncating mutations (Frequency, percentage) in TCGA cohort	Truncating mutations (Frequency, percentage) in GENIE cohort	Truncating mutations p-value (chi-square test)	Truncating mutations q-value	Inframe mutations (Frequency, percentage) in TCGA cohort	Inframe mutations (Frequency, percentage) in GENIE cohort	Inframe mutations p-value (chi-square test)	Inframe mutations q-value
**PIK3CA**	**156, 31.8**	**489, 28.4**	**0.1**	**0.6**	**2, 0.4**	**2, 0.1**	**0.179**	**0.7**	**7, 1.4**	**22, 1.3**	**0.8**	**0.82**
ERBB2	7, 1.4	23, 1.3	0.9	1.0	0, 0.0	0, 0.0	ns	ns	0, 0.0	3, 0.2	0.4	0.7
AKT1	16, 3.3	69, 4.0	0.5	0.8	0, 0.0	0, 0.0	ns	ns	0, 0.0	0, 0.0	ns	ns
RPTOR	**7, 1.4**	**4, 0.2**	***0.0009	**0.04**	**2, 0.4**	**0, 0.0**	**0.0080	0.2	0, 0.0	0, 0.0	ns	ns
KRAS	4, 0.8	11, 0.6	0.7	0.9	0, 0.0	0, 0.0	ns	ns	0, 0.0	0, 0.0	ns	ns
AKT3	0, 0.0	1, 0.1	0.6	0.9	0, 0.0	2, 0.1	0.4507	0.8	0, 0.0	0, 0.0	ns	ns
PIK3CB	3, 0.6	4, 0.2	0.2	0.6	1, 0.2	0, 0.0	0.0606	0.4	1, 0.2	0, 0.0	0.1	0.3
BRAF	1, 0.2	6, 0.3	0.6	0.9	1, 0.2	0, 0.0	0.0606	0.4	0, 0.0	1, 0.1	0.6	0.7
ERBB3	10, 2.0	25, 1.5	0.4	0.7	1, 0.2	2, 0.1	0.64	0.8	0, 0.0	0, 0.0	ns	ns
NF1	5, 1.0	22, 1.3	0.6	0.9	7, 1.4	22, 1.3	0.7934	0.9	3, 0.6	7, 0.4	0.5	0.7
FBXW7	5, 1.0	8, 0.5	0.2	0.6	1, 0.2	3, 0.2	0.89	1.0	0, 0.0	1, 0.1	0.6	0.7
EGFR	5, 1.0	18, 1.0	1.0	1.0	0, 0.0	1, 0.1	0.5939	0.8	0, 0.0	1, 0.1	0.6	0.7
IGF1	0, 0.0	4, 0.2	0.3	0.7	0, 0.0	0, 0.0	ns	ns	0, 0.0	0, 0.0	ns	ns
JAK2	3, 0.6	12, 0.7	0.8	1.0	1, 0.2	4, 0.2	0.9085	1.0	1, 0.2	0, 0.0	0.1	0.3
MAP2K1	1, 0.2	5, 0.3	0.7	0.9	1, 0.2	2, 0.1	0.64	0.8	0, 0.0	1, 0.1	0.6	0.7
TP53	133, 27.1	418, 24.2	0.2	0.6	70, 14.3	273, 15.8	0.4029	0.8	17, 3.5	49, 2.8	0.5	0.7
CDKN2B	0, 0.0	5, 0.3	0.2	0.6	0, 0.0	0, 0.0	ns	ns	0, 0.0	0, 0.0	ns	ns
CDK6	1, 0.2	2, 0.1	0.6	0.9	0, 0.0	0, 0.0	ns	ns	0, 0.0	0, 0.0	ns	ns
CCND1	1, 0.2	0, 0.0	0.1	0.3	0, 0.0	1, 0.1	0.5939	0.8	0, 0.0	1, 0.1	ns	ns
CCNE1	1, 0.2	3, 0.2	0.9	1.0	0, 0.0	1, 0.1	0.5939	0.8	0, 0.0	0, 0.0	ns	ns
CCND2	1, 0.2	1, 0.1	0.3	0.7	0, 0.0	1, 0.1	0.5939	0.8	0, 0.0	0, 0.0	ns	ns
CCND3	2, 0.4	3, 0.2	0.3	0.7	1, 0.2	0, 0.0	0.0606	0.4	0, 0.0	0, 0.0	ns	ns
CDK4	1, 0.2	4, 0.2	0.9	1.0	0, 0.0	0, 0.0	ns	ns	0, 0.0	0, 0.0	ns	ns
CDKN2A	**0, 0.0**	**14, 0.8**	*0.0454	0.3	1, 0.2	1, 0.1	0.3422	0.8	0, 0.0	1, 0.1	0.6	0.7
RB1	5, 1.0	15, 0.9	0.8	0.9	12, 2.4	25, 1.5	0.128	0.6	1, 0.2	5, 0.3	0.7	0.8
NOTCH4	**6, 1.2**	**5, 0.3**	**0.0094	0.1	1, 0.2	0, 0.0	0.0606	0.4	0, 0.0	0, 0.0	ns	ns
NOTCH1	**3, 0.6**	**33, 1.9**	*0.0444	0.3	2, 0.4	4, 0.2	0.5081	0.8	0, 0.0	1, 0.1	0.6	0.7
FGFR2	6, 1.2	11, 0.6	0.2	0.6	0, 0.0	0, 0.0	ns	ns	0, 0.0	0, 0.0	ns	ns
MET	4, 0.8	13, 0.8	0.9	1.0	0, 0.0	0, 0.0	ns	ns	0, 0.0	0, 0.0	ns	ns
FGFR1	1, 0.2	7, 0.4	0.5	0.8	0, 0.0	0, 0.0	ns	ns	0, 0.0	0, 0.0	ns	ns
GATA3	6, 1.2	20, 1.2	0.9	1.0	46, 9.4	154, 8.9	0.7565	0.9	**18, 3.7**	**6, 0.3**	****<0.0001	**0.0021**
CD274	0, 0.0	5, 0.3	0.2	0.6	0, 0.0	0, 0.0	ns	ns	0, 0.0	1, 0.1	0.6	0.7
ATM	8, 1.6	38, 2.2	0.4	0.8	4, 0.8	16, 0.9	0.8157	0.9	1, 0.2	1, 0.1	0.3	0.7
PALB2	2, 0.4	13, 0.8	0.4	0.8	1, 0.2	8, 0.5	0.4248	0.8	0, 0.0	0, 0.0	ns	ns
BRCA2	**4, 0.8**	**40, 2.3**	*0.0353	0.3	6, 1.2	16, 0.9	0.5594	0.8	2, 0.4	1, 0.1	0.1	0.3
BRCA1	**11, 2.2**	**16, 0.9**	*0.0191	0.2	2, 0.4	19, 1.1	0.162	0.7	1, 0.2	1, 0.1	0.3	0.7
BARD1	0, 0.0	2, 0.1	0.5	0.8	0, 0.0	1, 0.1	0.5939	0.8	0, 0.0	0, 0.0	ns	ns
AR	2, 0.4	9, 0.5	0.8	0.9	1, 0.2	1, 0.1	0.3422	0.8	1, 0.2	2, 0.1	0.6	0.7
ESR1	1, 0.2	19, 1.1	0.1	0.3	2, 0.4	4, 0.2	0.5081	0.8	1, 0.2	1, 0.1	0.3	0.7
PGR	**2, 0.4**	**0, 0.0**	**0.0080	0.1	0, 0.0	0, 0.0	ns	ns	0, 0.0	1, 0.1	0.6	0.7

*- significant p-value, ns-not significant

Supplementary Table S3: Percentages of individual mutation hotspots in the TCGA and GENIE ILC cohort and COSMIC IDs

Gene	Protein Change	No. of tumors affected in TCGA cohort	No. of tumors affected in GENIE cohort	Proportion of tumors affected in TCGA cohort	Proportion of tumors affected in GENIE cohort	COSMIC_IDs for hotspots for TCGA cohort	COSMIC_IDs for hotspots for GENIE cohort
PIK3CA	R88Q	1	0	0.79	0.00	746	
	Q546R	1	1	0.79	0.40	12459	12459
	N345K	4	13	3.15	5.24	754	754
	N345H	1	0	0.79	0.00	3846786	
	M1043V	1	0	0.79	0.00	12591	
	K111del	1	0	0.79	0.00	750	
	H1065L	1	0	0.79	0.00	17448	
	H1047R	25	47	19.69	18.95	775	775
	H1047L	3	1	2.36	0.40	776	776
	G118D	1	2	0.79	0.81	751	751
	E970K	1	0	0.79	0.00	94980	
	E726K	2	4	1.57	1.61	87306	87306
	E545R	1	0	0.79	0.00	Recurrent hotspot, http://cancerhotspots.org/ and http://3dhotspots.org/	
	E545K	19	23	14.96	9.27	763	763
	E542G	2	0	1.57	0.00	761	761
	C378F	1	0	0.79	0.00	21450	
	C378Y	0	1	0.00	0.40		1041479
	C420_P421del	0	2	0.00	0.81		Recurrent hotspot, http://cancerhotspots.org/ and http://3dhotspots.org/
	C420R	0	2	0.00	0.81		757
	E365K	0	1	0.00	0.40		86044
	E453del	0	1	0.00	0.40		Recurrent hotspot, http://cancerhotspots.org/ and http://3dhotspots.org/
	E453K	0	5	0.00	2.02		12584
	E542K	0	20	0.00	8.06		760
	E542V	0	1	0.00	0.40		762
	E545A	0	1	0.00	0.40		12458
	E545G	0	1	0.00	0.40		764
	E81K	0	1	0.00	0.40		27502
	G1049R	0	2	0.00	0.81		12597
	G364R	0	1	0.00	0.40		86042
	H450_L455del	0	1	0.00	0.40		Recurrent hotspot, http://cancerhotspots.org/ and http://3dhotspots.org/
	K111E	0	1	0.00	0.40		13570
	M1043I	0	2	0.00	0.81		29313
	M1043L	0	1	0.00	0.40		Recurrent hotspot, http://cancerhotspots.org/ and http://3dhotspots.org/
	N1044K	0	1	0.00	0.40		12592
	N107I	0	1	0.00	0.40		Recurrent hotspot, http://cancerhotspots.org/ and http://3dhotspots.org/
	N345I	0	1	0.00	0.40		94978
	P539R	0	1	0.00	0.40		759
	Q546K	0	2	0.00	0.81		766
	V105_G106del	0	1	0.00	0.40		Recurrent hotspot, http://cancerhotspots.org/ and http://3dhotspots.org/
	Y1021N	0	1	0.00	0.40		6143
AKT1	E17K	2	12	1.57	4.84	33765	33765
	D323G	0	1	0.00	0.40		1748728
KRAS	G12C	1	0	0.79	0.00		516
	G12S	1	0	0.79	0.00		517
	G13D	0	1	0.00	0.40		532
	G12A	0	1	0.00	0.40		522
ERBB2	L755S	1	10	0.79	4.03	14060	14060
	V777L	1	2	0.79	0.81	14062	14062
	R678Q	1	0	0.79	0.00	436498	
	L755R	1	0	0.79	0.00	Recurrent hotspot, http://cancerhotspots.org/ and http://3dhotspots.org/	
	I767M	1	0	0.79	0.00	51317	
	D769Y	0	1	0.00	0.40		1251412
	G776V	0	1	0.00	0.40		18609
	G778_P780dup	0	1	0.00	0.40		Recurrent hotspot, http://cancerhotspots.org/ and http://3dhotspots.org/
	L869R	0	2	0.00	0.81		249793
	LRENT755AQSQQ	0	1	0.00	0.40		Recurrent hotspot, http://cancerhotspots.org/ and http://3dhotspots.org/
	S310F	0	1	0.00	0.40		Recurrent hotspot, http://cancerhotspots.org/ and http://3dhotspots.org/
	Y772_A775dup	0	1	0.00	0.40		Recurrent hotspot, http://cancerhotspots.org/ and http://3dhotspots.org/
BRAF	D594N	0	1	0.00	0.40		27639
	G464V	0	1	0.00	0.40		450
ERBB3	E928G	0	5	0.00	2.02		1363010
	G284R	0	1	0.00	0.40		48360
	S846I	0	1	0.00	0.40		Recurrent hotspot, http://cancerhotspots.org/ and http://3dhotspots.org/
TP53	R273H	2	1	1.57	0.40	10660	10660
	L130V	1	0	0.79	0.00	11462	
	M246I	1	0	0.79	0.00	44310	
	P151A	1	0	0.79	0.00	44944	
	F341S	1	0	0.79	0.00	1386561	
	R280K	0	1	0.00	0.40		10728
	R175H	0	1	0.00	0.40		10648
	R337C	0	1	0.00	0.40		11071
	R249M	0	1	0.00	0.40		43871
	C135Y	0	1	0.00	0.40		10801
	E285K	0	1	0.00	0.40		10722
	Y220C	0	1	0.00	0.40		10758
	I251F	0	1	0.00	0.40		43967
	C277F	0	2	0.00	0.81		10749
	C238Y	0	1	0.00	0.40		11059
	Y107D	0	1	0.00	0.40		46103
GATA3	M293K	1	0	0.79	0.00	Recurrent hotspot, http://cancerhotspots.org/ and http://3dhotspots.org/	
ESR1	E380Q	1	2	0.79	0.81	3829320	3829320
	Y537S	0	1	0.00	0.40		3829320
	L536H	0	1	0.00	0.40		Recurrent hotspot, http://cancerhotspots.org/ and http://3dhotspots.org/

Supplementary Table S4: Percentages of individual mutation hotspots in the TCGA and GENIE IDC cohort and COSMIC IDs

Gene	Protein Change	No. of tumors affected in TCGA cohort	No. of tumors affected in GENIE cohort	Proportion of tumors affected in TCGA cohort	Proportion of tumors affected in GENIE cohort	COSMIC_IDs for hotspots for TCGA cohort	COSMIC_IDs for hotspots for GENIE cohort
**PIK3CA**	C378F	0	1	0.00	0.06		21450
	C420R	4	17	0.82	0.99	757	757
	C90*	0	1	0.00	0.06		Recurrent hotspot, http://cancerhotspots.org/ and http://3dhotspots.org/
	C901F	1	0	0.20	0.00	769	
	D1017E	0	1	0.00	0.06		1420961
	D1017H	0	3	0.00	0.17		27377
	D350G	1	0	0.20	0.00	271785	
	D350N	0	1	0.00	0.06		582511
	D725G	0	1	0.00	0.06		1420886
	D939G	0	1	0.00	0.06		1041513
	E103_G106delinsD	1	0	0.20	0.00	Recurrent hotspot, http://cancerhotspots.org/ and http://3dhotspots.org/	
	E103_P104del	1	0	0.20	0.00	Recurrent hotspot, http://cancerhotspots.org/ and http://3dhotspots.org/	
	E109_I112delinsD	1	0	0.20	0.00	Recurrent hotspot, http://cancerhotspots.org/ and http://3dhotspots.org/	
	E110del	0	3	0.00	0.17		Recurrent hotspot, http://cancerhotspots.org/ and http://3dhotspots.org/
	E365K	0	1	0.00	0.06		86044
	E365V	1	0	0.20	0.00	1484861	
	E453_L456del	0	1	0.00	0.06		Recurrent hotspot, http://cancerhotspots.org/ and http://3dhotspots.org/
	E453K	3	2	0.61	0.12	12584	12584
	E453Q	0	1	0.00	0.06		758
	E522K	0	1	0.00	0.06		28540
	E542A	0	1	0.00	0.06		1041495
	E542G	0	1	0.00	0.06		761
	E542K	19	60	3.88	3.48	760	760
	E542Q	0	2	0.00	0.12		17442
	E545A	1	3	0.20	0.17	12458	12458
	E545D	0	1	0.00	0.06		765
	E545G	0	1	0.00	0.06		764
	E545K	30	97	6.12	5.63	763	763
	E545Q	0	1	0.00	0.06		27133
	E726K	5	8	1.02	0.46	87306	87306
	E81K	2	3	0.41	0.17	27502	27502
	E970K	0	4	0.00	0.23		94980
	G1007R	2	1	0.41	0.06	17443	17443
	G1049R	1	7	0.20	0.41	12597	12597
	G1050S	0	1	0.00	0.06		249882
	G106_E109del	0	1	0.00	0.06		Recurrent hotspot, http://cancerhotspots.org/ and http://3dhotspots.org/
	G106_R108del	1	2	0.20	0.12	Recurrent hotspot, http://cancerhotspots.org/ and http://3dhotspots.org/	Recurrent hotspot, http://cancerhotspots.org/ and http://3dhotspots.org/
	G106R	0	1	0.00	0.06		86046
	G118D	2	3	0.41	0.17	751	751
	H1047L	4	37	0.82	2.15	776	776
	H1047Q	0	2	0.00	0.12		24714
	H1047R	66	183	13.47	10.61	775	775
	H1047Y	1	0	0.20	0.00	774	
	H1065Lfs*5	0	1	0.00	0.06		27132
	HH1047RR	0	2	0.00	0.12		Recurrent hotspot, http://cancerhotspots.org/ and http://3dhotspots.org/
	I1019V	0	1	0.00	0.06		249140
	I102_E103delinsK	0	2	0.00	0.12		Recurrent hotspot, http://cancerhotspots.org/ and http://3dhotspots.org/
	I1058L	0	1	0.00	0.06		479744
	I112F	0	1	0.00	0.06		Recurrent hotspot, http://cancerhotspots.org/ and http://3dhotspots.org/
	I112S	0	1	0.00	0.06		Recurrent hotspot, http://cancerhotspots.org/ and http://3dhotspots.org/
	K111_N114del	0	1	0.00	0.06		Recurrent hotspot, http://cancerhotspots.org/ and http://3dhotspots.org/
	K111del	0	1	0.00	0.06		750
	K111E	1	4	0.20	0.23	13570	13570
	K111N	1	0	0.20	0.00	12580	
	K711N	0	1	0.00	0.06		Recurrent hotspot, http://cancerhotspots.org/ and http://3dhotspots.org/
	L452_P458del	0	2	0.00	0.12		Recurrent hotspot, http://cancerhotspots.org/ and http://3dhotspots.org/
	M1004I	2	3	0.41	0.17	1420934	1420934
	M1043I	2	0	0.41	0.00	29313	
	M1043V	0	4	0.00	0.23		12591
	N1044K	0	4	0.00	0.23		12592
	N1044Y	2	0	0.41	0.00	36288	
	N114S	0	1	0.00	0.06		Recurrent hotspot, http://cancerhotspots.org/ and http://3dhotspots.org/
	N183S	0	1	0.00	0.06		Recurrent hotspot, http://cancerhotspots.org/ and http://3dhotspots.org/
	N345I	0	2	0.00	0.12		94978
	N345K	9	36	1.84	2.09	754	754
	N345T	1	0	0.20	0.00	446002	
	P104_N107delinsH	0	1	0.00	0.06		Recurrent hotspot, http://cancerhotspots.org/ and http://3dhotspots.org/
	P104_V105del	0	1	0.00	0.06		Recurrent hotspot, http://cancerhotspots.org/ and http://3dhotspots.org/
	P104L	1	1	0.20	0.06	163485	163485
	P104S	0	1	0.00	0.06		Recurrent hotspot, http://cancerhotspots.org/ and http://3dhotspots.org/
	P447_L455del	2	0	0.41	0.00	Recurrent hotspot, http://cancerhotspots.org/ and http://3dhotspots.org/	
	P449L	0	1	0.00	0.06		1041483
	P539R	1	3	0.20	0.17	759	759
	P97S	0	1	0.00	0.06		Recurrent hotspot, http://cancerhotspots.org/ and http://3dhotspots.org/
	Q546E	0	1	0.00	0.06		6147
	Q546H	0	1	0.00	0.06		24712
	Q546K	2	14	0.41	0.81	766	766
	Q546P	1	2	0.20	0.12	767	767
	Q546R	4	10	0.82	0.58	12459	12459
	Q958K	0	1	0.00	0.06		Recurrent hotspot, http://cancerhotspots.org/ and http://3dhotspots.org/
	R108C	0	1	0.00	0.06		Recurrent hotspot, http://cancerhotspots.org/ and http://3dhotspots.org/
	R108H	1	2	0.20	0.12	27497	27497
	R349Q	0	1	0.00	0.06		1420792
	R357Q	0	1	0.00	0.06		276751
	R88Q	1	0	0.20	0.00	746	
	R93G	0	1	0.00	0.06		Recurrent hotspot, http://cancerhotspots.org/ and http://3dhotspots.org/
	R93L	0	1	0.00	0.06		1420744
	R93Q	0	2	0.00	0.12		86041
	T1025A	0	1	0.00	0.06		771
	T1025I	0	1	0.00	0.06		36285
	V105_G106del	0	1	0.00	0.06		Recurrent hotspot, http://cancerhotspots.org/ and http://3dhotspots.org/
	V105_N107delinsG	0	1	0.00	0.06		Recurrent hotspot, http://cancerhotspots.org/ and http://3dhotspots.org/
	V105_R108del	1	2	0.20	0.12	Recurrent hotspot, http://cancerhotspots.org/ and http://3dhotspots.org/	Recurrent hotspot, http://cancerhotspots.org/ and http://3dhotspots.org/
	V346_N347dup	0	1	0.00	0.06		Recurrent hotspot, http://cancerhotspots.org/ and http://3dhotspots.org/
	W11_P18delinsGA	0	1	0.00	0.06		Recurrent hotspot, http://cancerhotspots.org/ and http://3dhotspots.org/
	Y1021C	0	2	0.00	0.12		12461
	Y1021H	0	2	0.00	0.12		17444
	Y355_G363dup	0	1	0.00	0.06		Recurrent hotspot, http://cancerhotspots.org/ and http://3dhotspots.org/
**AKT1**	E17K	15	16	3.06	0.93	33765	33765
	L52R	0	2	0.00	0.12		93893
	E17R	0	1	0.00	0.06		Recurrent hotspot, http://cancerhotspots.org/ and http://3dhotspots.org/
	L52H	0	1	0.00	0.06		Recurrent hotspot, http://cancerhotspots.org/ and http://3dhotspots.org/
	D323G	0	1	0.00	0.06		1748728
**FBXW7**	R465C	2	0	0.41	0.00	22932	
	R479Q	1	0	0.20	0.00	22974	
**KRAS**	G12V	3	4	0.61	0.23	520	520
	G12D	1	5	0.20	0.29	521	521
	G12A	0	1	0.00	0.06		522
**ERBB2**	D769Y	2	2	0.41	0.12	1251412	1251412
	D769H	1	0	0.20	0.00	13170	
	S310F	1	2	0.20	0.12	48358	48358
	L755S	0	1	0.00	0.06		48358
	V777L	0	2	0.00	0.12		14062
	R678Q	0	1	0.00	0.06		436498
	G778_P780dup	0	2	0.00	0.12		Recurrent hotspot, http://cancerhotspots.org/ and http://3dhotspots.org/
	Y772_A775dup	0	1	0.00	0.06		Recurrent hotspot, http://cancerhotspots.org/ and http://3dhotspots.org/
**CDKN2A**	H83Y	0	3	0.00	0.17		12504
	H83D	0	1	0.00	0.06		Recurrent hotspot, http://cancerhotspots.org/ and http://3dhotspots.org/
	D84G	0	1	0.00	0.06		13274
**FGFR2**	N549K	2	2	0.41	0.12	36912	
	Y375C	0	1	0.00	0.06		36904
	S252L	0	1	0.00	0.06		Recurrent hotspot, http://cancerhotspots.org/ and http://3dhotspots.org/
	P253R	0	1	0.00	0.06		Recurrent hotspot, http://cancerhotspots.org/ and http://3dhotspots.org/
	C382R	1	0	0.20	0.00	36906	
**GATA3**	M293K	1	3	0.20	0.17	Recurrent hotspot, http://cancerhotspots.org/ and http://3dhotspots.org/	Recurrent hotspot, http://cancerhotspots.org/ and http://3dhotspots.org/
	M293R	0	2	0.00	0.12		Recurrent hotspot, http://cancerhotspots.org/ and http://3dhotspots.org/
	R364T	0	1	0.00	0.06		Recurrent hotspot, http://cancerhotspots.org/ and http://3dhotspots.org/
	R364S	1	0	0.20	0.00	Recurrent hotspot, http://cancerhotspots.org/ and http://3dhotspots.org/	
**ESR1**	E380Q	0	3	0.00	0.17		3829320
	D538G	0	3	0.00	0.17		94250
	Y537S	0	3	0.00	0.17		1074639
	L536P	0	1	0.00	0.06		Recurrent hotspot, http://cancerhotspots.org/ and http://3dhotspots.org/
	R337H	0	2	0.00	0.12	21301	
	R337L	0	1	0.00	0.06		Recurrent hotspot, http://cancerhotspots.org/ and http://3dhotspots.org/
**ATM**	I2888T	0	1	0.00	0.06		21679
	R337C	0	1	0.00	0.06		21323
	N2875S	1	0	0.20	0.00	1183939	
	R220H	0	1	0.00	0.06		Recurrent hotspot, http://cancerhotspots.org/ and http://3dhotspots.org/
	E203K	1	0	0.20	0.00	232755	
**CDK6**	G128C	0	1	0.00	0.06		Recurrent hotspot, http://cancerhotspots.org/ and http://3dhotspots.org/
	N546K	0	1	0.00	0.06		19176
**MAP2K1**	G284R	1	0	0.20	0.00	48360	
**FGFR1**	D297Y	1	0	0.20	0.00	1299647	
**ERBB3**	E928G	0	4	0.00	0.23		1363010
	A232V	0	1	0.00	0.06		1242240
	V104L	0	1	0.00	0.06		1299642
	S846I	0	1	0.00	0.06		Recurrent hotspot, http://cancerhotspots.org/ and http://3dhotspots.org/
	K329E	0	1	0.00	0.06		Recurrent hotspot, http://cancerhotspots.org/ and http://3dhotspots.org/
**PIK3CB**	E1051K	1	0	0.20	0.00	317875	
**EGFR**	E746_A750del	0	1	0.00	0.06		Recurrent hotspot, http://cancerhotspots.org/ and http://3dhotspots.org/
	R222H	0	1	0.00	0.06		Recurrent hotspot, http://cancerhotspots.org/ and http://3dhotspots.org/
**TP53**	R248Q	3	26	0.61	1.51	10662	10662
	R175H	12	24	2.45	1.39	10648	10648
	R273H	5	24	1.02	1.39	10660	10660
	R248W	4	17	0.82	0.99	10656	10656
	R273C	3	14	0.61	0.81	10659	10659
	V173M	2	12	0.41	0.70	11084	11084
	R282W	1	9	0.20	0.52	10704	10704
	M237I	1	7	0.20	0.41	10834	10834
	Y163C	2	7	0.41	0.41	10808	10808
	C135Y	1	6	0.20	0.35	10801	10801
	E285K	2	6	0.41	0.35	10722	10722
	H179R	2	5	0.41	0.29	10889	10889
	H193R	5	5	1.02	0.29	10742	10742
	I195T	4	5	0.82	0.29	11089	11089
	L194R	2	5	0.41	0.29	44571	44571
	Y220C	4	5	0.82	0.29	10758	10758
	Y236C	0	5	0.00	0.29		10731
	G245S	1	4	0.20	0.23	6932	6932
	H193L	1	4	0.20	0.23	11066	11066
	P278S	0	4	0.00	0.23		10939
	R110P	0	4	0.00	0.23		11250
	R280T	2	4	0.41	0.23	10724	10724
	R337C	0	4	0.00	0.23		11071
	V173L	1	4	0.20	0.23	44057	44057
	Y126C	1	4	0.20	0.23	11517	11517
	C135W	1	3	0.20	0.17	44219	44219

	C176F	3	3	0.61	0.17	10645	10645
	C238Y	2	3	0.41	0.17	11059	11059
	C275F	0	3	0.00	0.17		10701
	C275Y	0	3	0.00	0.17		10893
	E198*	1	3	0.20	0.17	44241	44241
	G245C	0	3	0.00	0.17		11081
	H179Y	2	3	0.41	0.17	10768	10768
	P278R	0	3	0.00	0.17		10887
	R249W	0	3	0.00	0.17		43629
	S241Y	0	3	0.00	0.17		10935
	Y220D	0	3	0.00	0.17		11847
	A138P	0	2	0.00	0.12		11188
	C176W	0	2	0.00	0.12		11114
	C176Y	0	2	0.00	0.12		10687
	C238F	1	2	0.20	0.12	43778	43778
	C238G	0	2	0.00	0.12		46336
	C242R	0	2	0.00	0.12		11738
	D281E	1	2	0.20	0.12	43906	43906
	E286K	1	2	0.20	0.12	10726	10726
	F270L	0	2	0.00	0.12		44262
	G244C	0	2	0.00	0.12		11524
	G244D	0	2	0.00	0.12		10883
	G245D	2	2	0.41	0.12	43606	43606
	G266R	0	2	0.00	0.12		10794
	H179P	0	2	0.00	0.12		44218
	H179Q	0	2	0.00	0.12		44214
	I254S	0	2	0.00	0.12		45035
	I255F	1	2	0.20	0.12	43651	43651
	K132M	0	2	0.00	0.12		43592
	K132R	0	2	0.00	0.12		11582
	L111P	2	2	0.41	0.12	44045	44045
	L252del	0	2	0.00	0.12		44247
	M246I	0	2	0.00	0.12		10757
	P151H	1	2	0.20	0.12	11476	11476
	P151S	0	2	0.00	0.12		10905
	R248L	0	2	0.00	0.12		6549
	R249S	0	2	0.00	0.12		10817
	R282G	0	2	0.00	0.12		10992
	R337L	1	2	0.20	0.12	11411	11411
	S127Y	0	2	0.00	0.12		43970
	S241C	0	2	0.00	0.12		10709
	S241F	1	2	0.20	0.12	10812	10812
	V157F	1	2	0.20	0.12	10670	10670
	V172D	0	2	0.00	0.12		44229
	V272L	0	2	0.00	0.12		10859
	V274G	0	2	0.00	0.12		43945
	Y163D	0	2	0.00	0.12		44216
	Y205D	0	2	0.00	0.12		43844
	G266E	2	1	0.41	0.06	10867	10867
	K132E	2	1	0.41	0.06	10813	10813
	K132N	2	1	0.41	0.06	43963	43963
	R273L	2	1	0.41	0.06	10779	10779
	R280K	2	1	0.41	0.06	10728	10728
	Y234C	4	1	0.82	0.06	10725	10725
	C141Y	3	0	0.61	0.00	43708	
	Y220S	2	0	0.41	0.00	43850	
	V216M	2	0	0.41	0.00	10667	
	A138V	0	1	0.00	0.06		45016
	A159V	0	1	0.00	0.06		11148
	A161T	0	1	0.00	0.06		10739
	A276G	0	1	0.00	0.06		45695
	C141_Q144del	0	1	0.00	0.06		Recurrent hotspot, http://cancerhotspots.org/ and http://3dhotspots.org/
	C176S	0	1	0.00	0.06		44645
	C238R	0	1	0.00	0.06		44321
	C242G	0	1	0.00	0.06		44135
	C242W	0	1	0.00	0.06		11356
	C242Y	0	1	0.00	0.06		10646
	C275G	0	1	0.00	0.06		11501
	C275R	0	1	0.00	0.06		43902
	C277F	0	1	0.00	0.06		10749
	D208V	0	1	0.00	0.06		44249
	D281G	0	1	0.00	0.06		11232
	D281N	0	1	0.00	0.06		43596
	E258K	0	1	0.00	0.06		10988
	E271K	0	1	0.00	0.06		10719
	E286G	0	1	0.00	0.06		43565
	F109C	0	1	0.00	0.06		78686
	F113C	1	1	0.20	0.06	10717	
	F113P	0	1	0.00	0.06		Recurrent hotspot, http://cancerhotspots.org/ and http://3dhotspots.org/
	F134C	0	1	0.00	0.06		43949
	F134L	0	1	0.00	0.06		11319
	F134V	0	1	0.00	0.06		43941
	G244R	0	1	0.00	0.06		44221
	G244S	0	1	0.00	0.06		10941
	G245_P250delinsA	0	1	0.00	0.06		Recurrent hotspot, http://cancerhotspots.org/ and http://3dhotspots.org/
	G262V	0	1	0.00	0.06		11198
	G266V	0	1	0.00	0.06		10958
	G279E	0	1	0.00	0.06		43714
	H168R	0	1	0.00	0.06		43545
	H193Q	0	1	0.00	0.06		Recurrent hotspot, http://cancerhotspots.org/ and http://3dhotspots.org/
	H193Y	0	1	0.00	0.06		10672
	H214R	0	1	0.00	0.06		43687
	I195F	0	1	0.00	0.06		44633
	I251S	0	1	0.00	0.06		43829
	I255del	0	1	0.00	0.06		43694
	I255S	1	1	0.20	0.06	10788	10788
	K164E	1	1	0.20	0.06	10762	10762
	L111R	0	1	0.00	0.06		44570
	L130F	0	1	0.00	0.06		11449
	L130H	0	1	0.00	0.06		46114
	L194F	0	1	0.00	0.06		10995
	L194P	1	1	0.20	0.06	43827	43827
	L257P	0	1	0.00	0.06		43842
	L257R	0	1	0.00	0.06		44890
	M133K	0	1	0.00	0.06		11781
	M133R	0	1	0.00	0.06		43730
	M243_G244insA	0	1	0.00	0.06		Recurrent hotspot, http://cancerhotspots.org/ and http://3dhotspots.org/
	M243_P250del	0	1	0.00	0.06		Recurrent hotspot, http://cancerhotspots.org/ and http://3dhotspots.org/
	M246L	0	1	0.00	0.06		45992
	M246R	0	1	0.00	0.06		11376
	M246V	0	1	0.00	0.06		43555
	N239D	0	1	0.00	0.06		10777
	P128del	0	1	0.00	0.06		45310
	P151R	0	1	0.00	0.06		44003
	P177R	0	1	0.00	0.06		10651
	P278A	1	1	0.20	0.06	10814	10814
	P278T	1	1	0.20	0.06	43697	43697
	R110L	0	1	0.00	0.06		10716
	R158L	0	1	0.00	0.06		10714
	R158P	0	1	0.00	0.06		43615
	R174W	0	1	0.00	0.06		44782
	R175G	0	1	0.00	0.06		10870
	R181C	0	1	0.00	0.06		11090
	R249G	0	1	0.00	0.06		10668
	R267P	0	1	0.00	0.06		11392
	R280G	0	1	0.00	0.06		11123
	R280I	0	1	0.00	0.06		11287
	R282P	0	1	0.00	0.06		44306
	R337H	0	1	0.00	0.06		43882
	R342P	0	1	0.00	0.06		45276
	S127F	0	1	0.00	0.06		44226
	S127P	1	1	0.20	0.06	44687	44687
	S183_G187delinsC	0	1	0.00	0.06		Recurrent hotspot, http://cancerhotspots.org/ and http://3dhotspots.org/
	S215R	0	1	0.00	0.06		44979
	S240_S241del	0	1	0.00	0.06		Recurrent hotspot, http://cancerhotspots.org/ and http://3dhotspots.org/
	S241A	0	1	0.00	0.06		44224
	S241P	0	1	0.00	0.06		44578
	T125K	0	1	0.00	0.06		44073
	T125R	0	1	0.00	0.06		45243
	T284P	0	1	0.00	0.06		44352
	V157D	0	1	0.00	0.06		44329
	V218E	0	1	0.00	0.06		44317
	V274A	0	1	0.00	0.06		44393
	V274L	0	1	0.00	0.06		44443
	Y107D	1	1	0.20	0.06	46103	46103
	Y205C	0	1	0.00	0.06		43947
	Y205F	0	1	0.00	0.06		11351
	Y205H	0	1	0.00	0.06		43642
	Y205N	1	1	0.20	0.06	45685	45685
	Y234D	0	1	0.00	0.06		43768
	Y234H	0	1	0.00	0.06		11152
	Y234Q	0	1	0.00	0.06		Recurrent hotspot, http://cancerhotspots.org/ and http://3dhotspots.org/
	Y236_N239delinsF	0	1	0.00	0.06		Recurrent hotspot, http://cancerhotspots.org/ and http://3dhotspots.org/
	Y236H	0	1	0.00	0.06		44326
	C135F	1	0	0.20	0.00	10647	
	C135R	1	0	0.20	0.00	10684	
	C141R	1	0	0.20	0.00	43901	
	D208G	1	0	0.20	0.00	45796	
	D281V	1	0	0.20	0.00	45729	
	E258Q	1	0	0.20	0.00	10751	
	H168P	1	0	0.20	0.00	44808	
	I162_Y163delinsN	1	0	0.20	0.00	Recurrent hotspot, http://cancerhotspots.org/ and http://3dhotspots.org/	
	I232S	1	0	0.20	0.00	45045	
	K132T	1	0	0.20	0.00	43912	
	L111Q	1	0	0.20	0.00	44630	
	L265P	1	0	0.20	0.00	11011	
	N247I	1	0	0.20	0.00	43995	
	P278L	1	0	0.20	0.00	10863	
	Q136E	1	0	0.20	0.00	43767	
	R156del	1	0	0.20	0.00	45595	
	S215I	1	0	0.20	0.00	11450	
	T155P	1	0	0.20	0.00	10912	
	V157G	1	0	0.20	0.00	43903	
	V274F	1	0	0.20	0.00	10769	
	Y107C	1	0	0.20	0.00	44994	
	Y126N	1	0	0.20	0.00	44380	

Supplementary Table S5: Frequencies, percentages and p-values of gain, amplifications, hemizygous deletions and homozygous deletions in ILC in the TCGA and GENIE cohorts

Genes	Gain (Frequency, percentage) in TCGA cohort	Gain (Frequency, percentage) in GENIE cohort	Gain p-value (fisher's exact test)	Gain q-value	Amplification (Frequency, percentage) in TCGA cohort	Amplification (Frequency, percentage) in GENIE cohort	Amplification p-value (fisher's exact test)	Amplification q-value	Hemizygous Deletion (Frequency, percentage) in TCGA cohort	Hemizygous Deletion (Frequency, percentage) in GENIE cohort	Hemizygous Deletion p-value (fisher's exact test)	Hemizygous Deletion q-value	Homozygous Deletion (Frequency, percentage) in TCGA cohort	Homozygous Deletion (Frequency, percentage) in GENIE cohort	Homozygous Deletion p-value (fisher's exact test)	Homozygous Deletion q-value
FBXW7	1, 0.8	1, 0.5	>0.9	0.0758	0, 0.0	0, 0.0	>0.9	0.9974	21, 16.5	4, 1.9	****<0.0001	0.000035	0, 0.0	0, 0.0	>0.9	0.9999
MAP2K1	3, 2.4	0, 0.0	0.1	0.0118	0, 0.0	0, 0.0	>0.9	0.9974	18, 14.2	1, 0.5	****<0.0001	0.000035	0, 0.0	0, 0.0	>0.9	0.9999
PIK3CA	5, 3.9	1, 0.5	*0.0318	0.0071	2, 1.57	1, 0.5	0.6	0.9974	3, 2.4	0, 0.0	0.055	0.0115	0, 0.0	0, 0.0	>0.9	0.9999
PIK3CB	7, 5.5	0, 0.0	**0.0011	0.0004	1, 0.79	0, 0.0	0.4	0.9974	6, 4.7	0, 0.0	**0.0029	0.0008	0, 0.0	0, 0.0	>0.9	0.9999
AKT1	12, 9.4	3, 1.5	**0.0015	0.0005	0, 0.0	0, 0.0	>0.9	0.9974	21, 16.5	3, 1.5	****<0.0001	0.000035	0, 0.0	0, 0.0	>0.9	0.9999
IGF1	13, 10.2	0, 0.0	****<0.0001	0.000038684	1, 0.8	0, 0.0	0.4	0.9974	12, 9.4	0, 0.0	****<0.0001	0.000035	0, 0.0	0, 0.0	>0.9	0.9999
ERBB2	14, 11.0	7, 3.4	**0.0091	0.0024	9, 7.1	7, 3.4	0.2	0.9974	26, 20.5	4, 1.9	****<0.0001	0.000035	0, 0.0	0, 0.0	>0.9	0.9999
JAK2	15, 11.8	0, 0.0	****<0.0001	0.000038684	0, 0.0	0, 0.0	>0.9	0.9974	11, 8.7	3, 1.5	**0.0031	0.0008	0, 0.0	0, 0.0	>0.9	0.9999
NF1	17, 13.4	3, 1.5	****<0.0001	0.000038684	0, 0.0	0, 0.0	>0.9	0.9974	31, 24.4	7, 3.4	****<0.0001	0.000035	0, 0.0	2, 0.97	0.5	0.5267
ERBB3	18, 14.2	12, 5.8	*0.0167	0.0042	1, 0.8	0, 0.0	0.4	0.9974	4, 3.1	0, 0.0	*0.0205	0.0049	0, 0.0	0, 0.0	>0.9	0.9999
KRAS	18, 14.2	3, 1.5	****<0.0001	0.000038684	0, 0.0	0, 0.0	>0.9	0.9974	5, 3.9	2, 1.0	0.1105	0.0226	0, 0.0	0, 0.0	>0.9	0.9999
RPTOR	19, 15.0	0, 0.0	****<0.0001	0.000038684	1, 0.8	0, 0.0	0.4	0.9974	25, 19.7	0, 0.0	****<0.0001	0.000035	0, 0.0	0, 0.0	>0.9	0.9999
BRAF	22, 17.3	2, 1.0	****<0.0001	0.000038684	0, 0.0	0, 0.0	>0.9	0.9974	9, 7.1	1, 0.5	***0.0010	0.0003	0, 0.0	0, 0.0	>0.9	0.9999
EGFR	26, 20.5	1, 0.5	****<0.0001	0.000038684	0, 0.0	0, 0.0	>0.9	0.9974	1, 0.8	2, 1.0	>0.9999	0.1837	0, 0.0	0, 0.0	>0.9	0.9999
AKT3	84, 66.1	47, 22.8	****<0.0001	0.000038684	21, 16.5	1, 0.5	****<0.0001	0.0020	1, 0.8	0, 0.0	0.4	0.0719	0, 0.0	0, 0.0	>0.9	0.9999
RB1	0, 0.0	0, 0.0	>0.9	0.1837	0, 0.0	0, 0.0	>0.9	0.9974	40, 31.5	15, 7.3	****<0.0001	0.000035	2, 1.6	0, 0.0	0.1	0.1447
TP53	5, 3.9	0, 0.0	**0.0077	0.0021	0, 0.0	0, 0.0	>0.9	0.9974	69, 54.3	27, 13.1	****<0.0001	0.000035	0, 0.0	0, 0.0	>0.9	0.9999
CCND3	10, 7.9	1, 0.5	**0.0077	0.0021	0, 0.0	0, 0.0	>0.9	0.9974	19, 15.0	1, 0.5	****<0.0001	0.000035	1, 0.8	0, 0.0	0.4	0.3814
CDKN2B	12, 9.4	1, 0.5	****<0.0001	0.000038684	1, 0.8	0, 0.0	0.4	0.9974	11, 8.7	2, 1.0	***0.0007	0.0002	1, 0.8	1, 0.5	>0.9	0.9999
CDKN2A	12, 9.4	1, 0.5	****<0.0001	0.000038684	1, 0.8	0, 0.0	0.4	0.9974	11, 8.7	2, 1.0	***0.0007	0.0002	1, 0.8	0, 0.0	0.4	0.3814
CCND1	14, 11.0	15, 7.3	0.3	0.0665	22, 17.3	35, 17.0	>0.9	0.9974	13, 10.2	1, 0.5	****<0.0001	0.000035	0, 0.0	0, 0.0	>0.9	0.9999
CCND2	16, 12.6	2, 1.0	****<0.0001	0.000038684	0, 0.0	0, 0.0	>0.9	0.9974	7, 5.5	6, 2.9	0.3	0.0493	1, 0.8	0, 0.0	0.4	0.3814
CCNE1	17, 13.4	2, 1.0	****<0.0001	0.000038684	0, 0.0	0, 0.0	0.4	0.9974	5, 3.9	0, 0.0	**0.0077	0.0019	0, 0.0	0, 0.0	>0.9	0.9999
CDK4	19, 15.0	7, 3.4	***0.0002	0.0001	0, 0.0	0, 0.0	>0.9	0.9974	3, 2.4	0, 0.0	0.055	0.0115	0, 0.0	0, 0.0	>0.9	0.9999
CDK6	23, 18.1	2, 1.0	****<0.0001	0.000038684	0, 0.0	0, 0.0	>0.9	0.9974	7, 5.5	2, 1.0	*0.0299	0.0067	0, 0.0	0, 0.0	>0.9	0.9999
FGFR2	8, 6.3	1, 0.5	**0.0024	0.0007	1, 0.8	0, 0.0	**0.0039	0.0519	18, 14.2	1, 0.5	****<0.0001	0.000035	0, 0.0	0, 0.0	>0.9	0.9999
NOTCH4	10, 7.9	0, 0.0	****<0.0001	0.000038684	0, 0.0	0, 0.0	>0.9	0.9974	15, 11.8	0, 0.0	****<0.0001	0.000035	0, 0.0	0, 0.0	>0.9	0.9999
NOTCH1	13, 10.2	2, 1.0	***0.0001	0.000038684	0, 0.0	0, 0.0	>0.9	0.9974	11, 8.7	3, 1.5	**0.0031	0.0008	0, 0.0	0, 0.0	>0.9	0.9999
MET	22, 17.3	2, 1.0	****<0.0001	0.000038684	0, 0.0	0, 0.0	>0.9	0.9974	8, 6.3	3, 1.5	*0.0244	0.0056	0, 0.0	0, 0.0	>0.9	0.9999
FGFR1	29, 22.8	2, 1.0	****<0.0001	0.000038684	12, 9.4	16, 7.8	0.7	0.9974	13, 10.2	11, 5.3	0.1	0.0249	0, 0.0	0, 0.0	>0.9	0.9999
CD274	14, 11.0	0, 0.0	****<0.0001	0.000038684	0, 0.0	0, 0.0	>0.9	0.9974	12, 9.4	3, 1.5	**0.0015	0.0004	0, 0.0	0, 0.0	>0.9	0.9999
GATA3	15, 11.8	26, 12.6	0.9	0.1631	1, 0.8	1, 0.5	>0.9	0.9974	11, 8.7	0, 0.0	****<0.0001	0.000035	0, 0.0	0, 0.0	>0.9	0.9999
BRCA2	1, 0.8	1, 0.5	0.4	0.0758	0, 0.0	1, 0.5	>0.9	0.9974	38, 29.9	12, 5.8	****<0.0001	0.000035	1, 0.8	1, 0.5	0.4	0.3814
BARD1	4, 3.1	0, 0.0	*0.0205	0.0047	0, 0.0	0, 0.0	>0.9	0.9974	14, 11.0	0, 0.0	****<0.0001	0.000035	0, 0.0	0, 0.0	>0.9	0.9999
ATM	4, 3.1	0, 0.0	*0.0205	0.0047	0, 0.0	29, 14.1	****<0.0001	0.0020	58, 45.7	0, 0.0	****<0.0001	0.000035	1, 0.8	0, 0.0	0.4	0.3814
BRCA1	14, 11.0	3, 1.5	***0.0002	0.0001	3, 2.4	0, 0.0	0.055	0.4365	33, 26.0	5, 2.4	****<0.0001	0.000035	0, 0.0	0, 0.0	>0.9	0.9999
PALB2	58, 45.7	20, 9.7	****<0.0001	0.000038684	4, 3.1	0, 0.0	*0.0205	0.2045	7, 5.5	0, 0.0	**0.0011	0.0003	0, 0.0	0, 0.0	>0.9	0.9999
PGR	4, 3.1	0, 0.0	*0.0205	0.0047	0, 0.0	0, 0.0	>0.9	0.9974	54, 42.5	0, 0.0	****<0.0001	0.000035	1, 0.8	0, 0.0	0.4	0.3814
ESR1	6, 4.7	8, 3.9	0.8	0.1511	1, 0.8	1, 0.5	>0.9	0.9974	29, 22.8	5, 2.4	****<0.0001	0.000035	1, 0.8	0, 0.0	0.4	0.3814
AR	8, 6.3	0, 0.0	***0.0004	0.0001	0, 0.0	1, 0.5	>0.9	0.9974	10, 7.9	0, 0.0	****<0.0001	0.000035	0, 0.0	0, 0.0	>0.9	0.9999
*- significant p-value.																

Supplementary Table S6: Frequencies, percentages and p-values of gain, amplifications, hemizygous deletions and homozygous deletions in IDC in the TCGA and GENIE cohorts

Genes	Gain (Frequency, percentage) in TCGA cohort	Gain (Frequency, percentage) in GENIE cohort	Gain p-value (fisher's exact test)	Gain q-value	Amplification (Frequency, percentage) in TCGA cohort	Amplification (Frequency, percentage) in GENIE cohort	Amplification p-value (fisher's exact test)	Amplification q-value	Hemizygous Deletion (Frequency, percentage) in TCGA cohort	Hemizygous Deletion (Frequency, percentage) in GENIE cohort	Hemizygous Deletion p-value (fisher's exact test)	Hemizygous Deletion q-value	Homozygous Deletion (Frequency, percentage) in TCGA cohort	Homozygous Deletion (Frequency, percentage) in GENIE cohort	Homozygous Deletion p-value (fisher's exact test)	Homozygous Deletion q-value
FBXW7	56, 11.4	18, 18.4	****<0.0001	0.0001	6, 1.2	3, 0.2	*0.0157	0.0126	168, 34.3	50, 3.8	****<0.00012	0.00013	6, 1.24	0, 0.0	***0.0004	0.0035
MAP2K1	62, 12.7	28, 2.2	****<0.0001	0.0001	6, 1.2	0, 0.0	***0.0004	0.0005	178, 36.3	21, 1.6	****<0.0001	0.0001	1, 0.2	0, 0.0	0.3	0.4398
PIK3CA	159, 32.4	61, 4.7	****<0.0001	0.0001	35, 7.1	12, 0.9	****<0.0001	0.0002	24, 4.9	10, 0.8	**0.0021	0.0021	1, 0.2	0, 0.0	0.3	0.4398
PIK3CB	143, 29.2	0, 0.0	****<0.0001	0.0001	12, 2.4	3, 0.2	****<0.0001	0.0002	43, 8.8	0, 0.0	****<0.0001	0.0001	0, 0.0	0, 0.0	1.0	0.8837
AKT1	80, 16.3	36, 2.8	****<0.0001	0.0001	8, 1.6	4, 0.3	**0.0050	0.0046	161, 32.9	35, 2.7	****<0.0001	0.0001	2, 0.4	0, 0.0	0.1	0.1763
IGF1	95, 19.4	0, 0.0	****<0.0001	0.0001	0, 0.0	1, 0.1	0.9999	0.4797	102, 20.8	0, 0.0	****<0.0001	0.0001	1, 0.2	0, 0.0	0.3	0.4398
ERBB2	99, 20.2	58, 4.5	****<0.0001	0.0001	73, 14.9	183, 14.1	0.7	0.3284	139, 28.4	33, 2.5	****<0.0001	0.0001	1, 0.2	0, 0.0	0.3	0.4398
JAK2	85, 17.3	42, 3.2	****<0.0001	0.0001	16, 3.3	10, 0.8	***0.0002	0.0003	172, 35.1	64, 4.9	****<0.0001	0.0001	5, 1.0	4, 6.3	0.1	0.1763
NF1	100, 20.4	34, 2.6	****<0.0001	0.0001	20, 4.1	3, 0.2	****<0.0001	0.0002	182, 37.1	54, 4.2	****<0.0001	0.0001	1, 0.2	10, 0.8	0.3	0.4528
ERBB3	111, 22.7	43, 3.3	****<0.0001	0.0001	2, 0.4	3, 0.2	0.6	0.3206	82, 16.7	12, 0.9	****<0.0001	0.0001	0, 0.0	0, 0.0	1.0	0.8837
KRAS	131, 26.7	42, 3.2	****<0.0001	0.0001	14, 2.9	15, 1.2	*0.0187	0.0136	58, 11.8	18, 1.4	****<0.0001	0.0001	2, 0.4	0, 0.0	0.1	0.1763
RPTOR	172, 35.1	5, 0.4	****<0.0001	0.0001	37, 7.6	4, 0.3	****<0.0001	0.0002	86, 17.6	3, 0.2	****<0.0001	0.0001	1, 0.2	0, 0.0	0.3	0.4398
BRAF	139, 28.4	51, 3.9	****<0.0001	0.0001	9, 1.8	4, 0.3	**0.0019	0.002	76, 15.5	27, 2.1	****<0.0001	0.0001	1, 0.2	0, 0.0	0.3	0.4398
EGFR	165, 33.7	28, 2.2	****<0.0001	0.0001	10, 2.0	19, 1.5	0.4	0.227	44, 9.0	29, 2.2	****<0.0001	0.0001	2, 0.4	1, 0.1	0.2	0.4056
AKT3	288, 58.8	165, 12.7	****<0.0001	0.0001	67, 13.7	9, 0.7	****<0.0001	0.0002	15, 3.1	6, 0.5	****<0.0001	0.0001	0, 0.0	0, 0.0	1.0	0.8837
RB1	45, 9.2	14, 1.1	****<0.0001	0.0001	2, 0.4	0, 0.0	0.07	0.0449	228, 46.5	91, 7.0	****<0.0001	0.0001	28, 5.7	15, 1.2	****<0.0001	0.0018
TP53	37, 7.6	6, 0.5	****<0.0001	0.0001	0, 0.0	0, 0.0	0.9999	0.4797	313, 63.9	157, 12.1	****<0.0001	0.0001	8, 1.6	5, 0.4	**0.0100	0.0442
CCND3	115, 23.5	30, 2.3	****<0.0001	0.0001	13, 2.7	5, 0.4	****<0.0001	0.0002	86, 17.6	11, 0.8	****<0.0001	0.0001	0, 0.0	1, 0.1	1.0	0.8837
CDKN2B	71, 14.5	49, 3.8	****<0.0001	0.0001	5, 1.0	2, 0.2	*0.0191	0.0136	174, 35.5	52, 4.0	****<0.0001	0.0001	20, 4.1	39, 3.0	0.3	0.4528
CDKN2A	72, 14.7	47, 3.6	****<0.0001	0.0001	6, 1.2	2, 0.2	**0.0068	0.0057	172, 35.1	51, 3.9	****<0.0001	0.0001	20, 4.1	41, 3.2	0.4	0.5377
CCND1	130, 26.5	104, 8.0	****<0.0001	0.0001	79, 16.1	189, 14.5	0.4	0.227	56, 11.4	9, 0.7	****<0.0001	0.0001	0, 0.0	0, 0.0	1.0	0.8837
CCND2	116, 23.7	34, 2.6	****<0.0001	0.0001	18, 3.7	9, 0.7	*0.0267	0.0177	82, 16.7	40, 3.1	****<0.0001	0.0001	1, 0.2	2, 0.2	1.0	0.8837
CCNE1	118, 24.1	41, 3.2	****<0.0001	0.0001	30, 6.1	23, 1.8	****<0.0001	0.0002	80, 16.3	13, 1.0	****<0.0001	0.0001	1, 0.2	0, 0.0	1.0	0.8837
CDK4	105, 21.4	29, 2.2	****<0.0001	0.0001	13, 2.7	14, 1.1	*0.0268	0.0177	70, 14.3	13, 1.0	****<0.0001	0.0001	0, 0.0	0, 0.0	1.0	0.8837
CDK6	143, 29.2	42, 3.2	****<0.0001	0.0001	10, 2.0	6, 0.5	**0.0033	0.0032	63, 12.9	18, 1.4	****<0.0001	0.0001	0, 0.0	0, 0.0	1.0	0.8837
FGFR2	34, 6.9	13, 1.0	****<0.0001	0.0001	12, 2.4	15, 1.2	0.052	0.032	151, 30.8	45, 3.5	****<0.0001	0.0001	0, 0.0	1, 0.1	1.0	0.8837
NOTCH4	125, 25.5	0, 0.0	****<0.0001	0.0001	10, 2.0	3, 0.2	***0.0003	0.0004	84, 17.1	0, 0.0	****<0.0001	0.0001	0, 0.0	1, 0.1	1.0	0.8837
NOTCH1	74, 15.1	24, 1.8	****<0.0001	0.0001	8, 1.6	1, 0.1	***0.0002	0.0003	153, 31.2	43, 3.3	****<0.0001	0.0001	3, 0.6	1, 0.1	0.1	0.1763
MET	137, 28.0	49, 3.8	****<0.0001	0.0001	5, 1.0	0, 0.0	**0.0015	0.0017	79, 16.1	27, 2.1	****<0.0001	0.0001	0, 0.0	0, 0.0	1.0	0.8837
FGFR1	127, 25.9	78, 6.0	****<0.0001	0.0001	68, 13.9	131, 10.1	*0.0281	0.018	129, 26.3	72, 5.5	****<0.0001	0.0001	7, 1.4	6, 0.5	0.1	0.1763
CD274	83, 16.9	40, 3.1	****<0.0001	0.0001	17, 3.5	7, 0.5	****<0.0001	0.0002	172, 35.1	64, 4.9	****<0.0001	0.0001	5, 1.0	1, 0.1	**0.0070	0.0354
GATA3	138, 28.2	168, 12.9	****<0.0001	0.0001	28, 5.7	27, 2.1	***0.0002	0.0003	57, 11.6	3, 0.2	****<0.0001	0.0001	0, 0.0	0, 0.0	1.0	0.8837
BRCA2	58, 11.8	26, 2.0	****<0.0001	0.0001	5, 1.0	2, 0.2	*0.0191	0.0136	216, 44.1	64, 4.9	****<0.0001	0.0001	9, 1.8	3, 0.2	***0.0008	0.0057
BARD1	46, 9.4	1, 0.1	****<0.0001	0.0001	4, 0.8	0, 0.0	**0.0056	0.0049	131, 26.7	2, 0.2	****<0.0001	0.0001	5, 1.0	0, 0.0	**0.0015	0.0088
ATM	60, 12.2	7, 0.5	****<0.0001	0.0001	2, 0.4	2, 0.2	0.3	0.1757	236, 48.2	128, 9.8	****<0.0001	0.0001	6, 1.2	0, 0.0	***0.0004	0.0035
BRCA1	98, 20.0	35, 2.7	****<0.0001	0.0001	10, 2.0	4, 0.3	***0.0007	0.0008	191, 39.0	64, 4.9	****<0.0001	0.0001	4, 0.8	2, 0.2	0.051	0.1763
PALB2	241, 49.2	90, 6.9	****<0.0001	0.0001	25, 5.1	1, 0.1	****<0.0001	0.0002	48, 9.8	4, 0.3	****<0.0001	0.0001	0, 0.0	0, 0.0	1.0	0.8837
PGR	71, 14.5	0, 0.0	****<0.0001	0.0001	4, 0.8	6, 0.5	0.5	0.2535	210, 42.9	0, 0.0	****<0.0001	0.0001	7, 1.4	0, 0.0	***0.0001	0.0018
ESR1	74, 15.1	32, 2.5	****<0.0001	0.0001	17, 3.5	5, 0.4	****<0.0001	0.0002	161, 32.9	61, 4.7	****<0.0001	0.0001	1, 0.2	1, 0.1	0.5	0.6427
AR	92, 18.8	7, 0.5	****<0.0001	0.0001	7, 1.4	2, 0.2	**0.0023	0.0023	87, 17.8	5, 0.4	****<0.0001	0.0001	2, 0.4	0, 0.0	0.07	0.1763

*- significant p-value.

Supplementary Table S7: Number of breast cancer patients analyzed by PCR and capture-based approach in the GENIE dataset

	**ILC**		**IDC**	
**Platform Used**	Mutations (n=248)	CNAs (n=248)	Mutations (n=1724)	CNAs (n=1724)
PCR	42	0	424	0
Hybridization capture	206	206	1300	1300

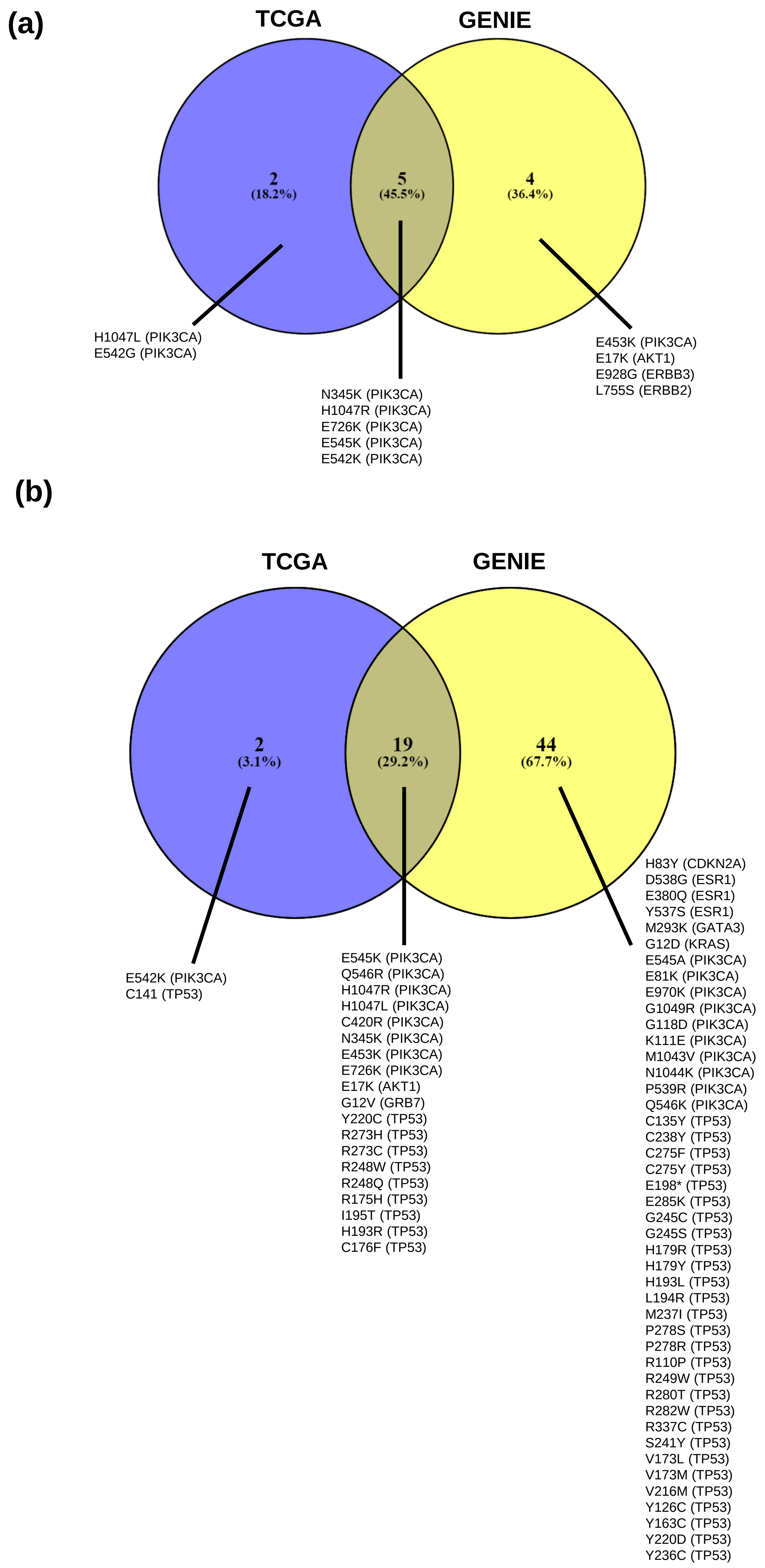
(a)
TCGA
GENIE
2
(18.2%)
5
(45.5%)
4
(36.4%)
H1047L (PIK3CA)
E542G (PIK3CA)
N345K (PIK3CA)
H1047R (PIK3CA)
E726K (PIK3CA)
E545K (PIK3CA)
E542K (PIK3CA)
E453K (PIK3CA)
E17K (AKT1)
E928G (ERBB3)
L755S (ERBB2)
(b)
TCGA
GENIE
2
(3.1%)
19
(29.2%)
44
(67.7%)
E542K (PIK3CA)
C141 (TP53)
E545K (PIK3CA)
Q546R (PIK3CA)
H1047R (PIK3CA)
H1047L (PIK3CA)
C420R (PIK3CA)
N345K (PIK3CA)
E453K (PIK3CA)
E726K (PIK3CA)
E17K (AKT1)
G12V (GRB7)
Y220C (TP53)
R273H (TP53)
R273C (TP53)
R248W (TP53)
R248Q (TP53)
R175H (TP53)
I195T (TP53)
H193R (TP53)
C176F (TP53)
H83Y (CDKN2A)
D538G (ESR1)
E380Q (ESR1)
Y537S (ESR1)
M293K (GATA3)
G12D (KRAS)
E545A (PIK3CA)
E81K (PIK3CA)
E970K (PIK3CA)
G1049R (PIK3CA)
G118D (PIK3CA)
K111E (PIK3CA)
M1043V (PIK3CA)
N1044K (PIK3CA)
P539R (PIK3CA)
Q546K (PIK3CA)
C135Y (TP53)
C238Y (TP53)
C275F (TP53)
C275Y (TP53)
E198* (TP53)
E285K (TP53)
G245C (TP53)
G245S (TP53)
H179R (TP53)
H179Y (TP53)
H193L (TP53)
L194R (TP53)
M237I (TP53)
P278S (TP53)
P278R (TP53)
R110P (TP53)
R249W (TP53)
R280T (TP53)
R282W (TP53)
R337C (TP53)
S241Y (TP53)
V173L (TP53)
V173M (TP53)
V216M (TP53)
Y126C (TP53)
Y163C (TP53)
Y220D (TP53)
Y236C (TP53)

Supplementary Legends:

Supplementary Fig. S1: Common and distinct mutational hotspots in the TCGA and GENIE cohorts across ILC and IDC. (a) Venn diagram of common and distinct mutational hotspots (>2) ILC patients in the TCGA and GENIE datasets. **(b)** Venn diagram of common and distinct mutational hotspots (>2) IDC patients in the TCGA and GENIE datasets.

Supplementary Table S1: Frequencies, percentages and p-values of missense, truncating and inframe mutations in ILC in the TCGA and GENIE cohorts

Supplementary Table S2: Frequencies, percentages and p-values of missense, truncating and inframe mutations in IDC in the TCGA and GENIE cohorts

Supplementary Table S3: Percentages of individual mutation hotspots in the TCGA and GENIE ILC cohort and COSMIC IDs

Supplementary Table S4: Percentages of individual mutation hotspots in the TCGA and GENIE IDC cohort and COSMIC IDs

Supplementary Table S5: Frequencies, percentages and p-values of gain, amplifications, hemizygous deletions and homozygous deletions in ILC in the TCGA and GENIE cohorts

Supplementary Table S6: Frequencies, percentages and p-values of gain, amplifications, hemizygous deletions and homozygous deletions in IDC in the TCGA and GENIE cohorts

Supplementary Table S7: Number of breast cancer patients analyzed by PCR and capture-based approach in GENIE dataset
